# Metabolic dependency profiling guides staggered antibiotic administration to enhance killing of antibiotic-tolerant *Acinetobacter baumannii*

**DOI:** 10.3389/fmicb.2026.1853821

**Published:** 2026-10-29

**Authors:** Ping Tuo, Yuanye Ji, Chunyan Ruan, Sijia Zhang, Fajiu Wang, Jia Shen, Xiaoming Xu, Peng Zhu

**Affiliations:** 1 Centre for Medical Research, Ningbo No. 2 Hospital, Ningbo, China; 2 Department of Thoracic Surgery, Ningbo No. 2 Hospital, Ningbo, China; 3 Department of Hepatopancreatobiliary Surgery Dept.1, Ningbo No. 2 Hospital, Ningbo, China; 4 Department of Urology, Ningbo No. 2 Hospital, Ningbo, China

**Keywords:** *Acinetobacter baumannii*, antibiotic tolerance, Metabolic Dependency Index, nutrient limitation, staggered administration

## Abstract

**Background:**

*Acinetobacter baumannii* infections are difficult to eradicate, and bacterial physiological state influences antibiotic killing. However, antibiotic-specific activity across nutrient-associated physiological states and quantitative comparison of these differences remain insufficiently understood.

**Methods:**

*Acinetobacter baumannii* ATCC 19606^T^ was cultured across a nutrient gradient (0–100% LB) to generate nutrient-associated physiological states. Bacterial growth, CFU-normalized intracellular ATP levels, and killing by six antibiotics were quantified. ATP–survival relationships were assessed across standardized concentrations of 0.5×−64×MIC, and a Metabolic Dependency Index (MDI) was developed as a continuous empirical measure of ATP-associated variation in antibiotic killing. Meropenem (MEM) and amikacin (AMK), representing opposite ends of the observed MDI spectrum, were evaluated using simultaneous, MEM-first staggered, and AMK-first staggered administration. The MEM–AMK combination was also examined in five clinical isolates.

**Results:**

Nutrient limitation reduced bacterial growth and CFU-normalized intracellular ATP levels and produced antibiotic- and concentration-dependent changes in killing. MDI values differed among antibiotics, with meropenem showing the highest and amikacin the lowest MDI. Simultaneous MEM–AMK treatment enhanced killing compared with the corresponding monotherapies under nutrient-limited conditions. MEM-first staggered administration further reduced bacterial counts relative to simultaneous treatment by 1.21 log_10_ CFU (95% CI, 0.48–1.94; *p* = 0.019) and 0.86 log_10_ CFU (95% CI, 0.47–1.26; *p* = 0.011) at two prespecified concentration combinations. Reverse-order AMK-first staggered treatment produced killing comparable to or greater than that achieved with MEM-first staggered treatment under several concentration combinations, indicating that the benefit of staggered exposure was not specific to a predefined MEM-first sequence. Similar differences between simultaneous and staggered MEM–AMK administration were observed across five clinical isolates, although the magnitude of these differences varied among isolates. Meropenem and amikacin MICs remained unchanged after antibiotic-free recovery of surviving populations, supporting a tolerance-associated phenotype rather than stable resistance.

**Conclusion:**

Antibiotic killing in *A. baumannii* varies across nutrient-associated physiological states in an antibiotic- and concentration-dependent manner. The MDI provides a continuous framework for comparing these differences, while the MEM–AMK experiments show that combination activity can be further influenced by antibiotic administration pattern. These findings provide a basis for integrating bacterial physiological state into the evaluation and optimization of antibiotic combinations under nutrient-limited conditions.

## Graphical abstract

**Figure:**
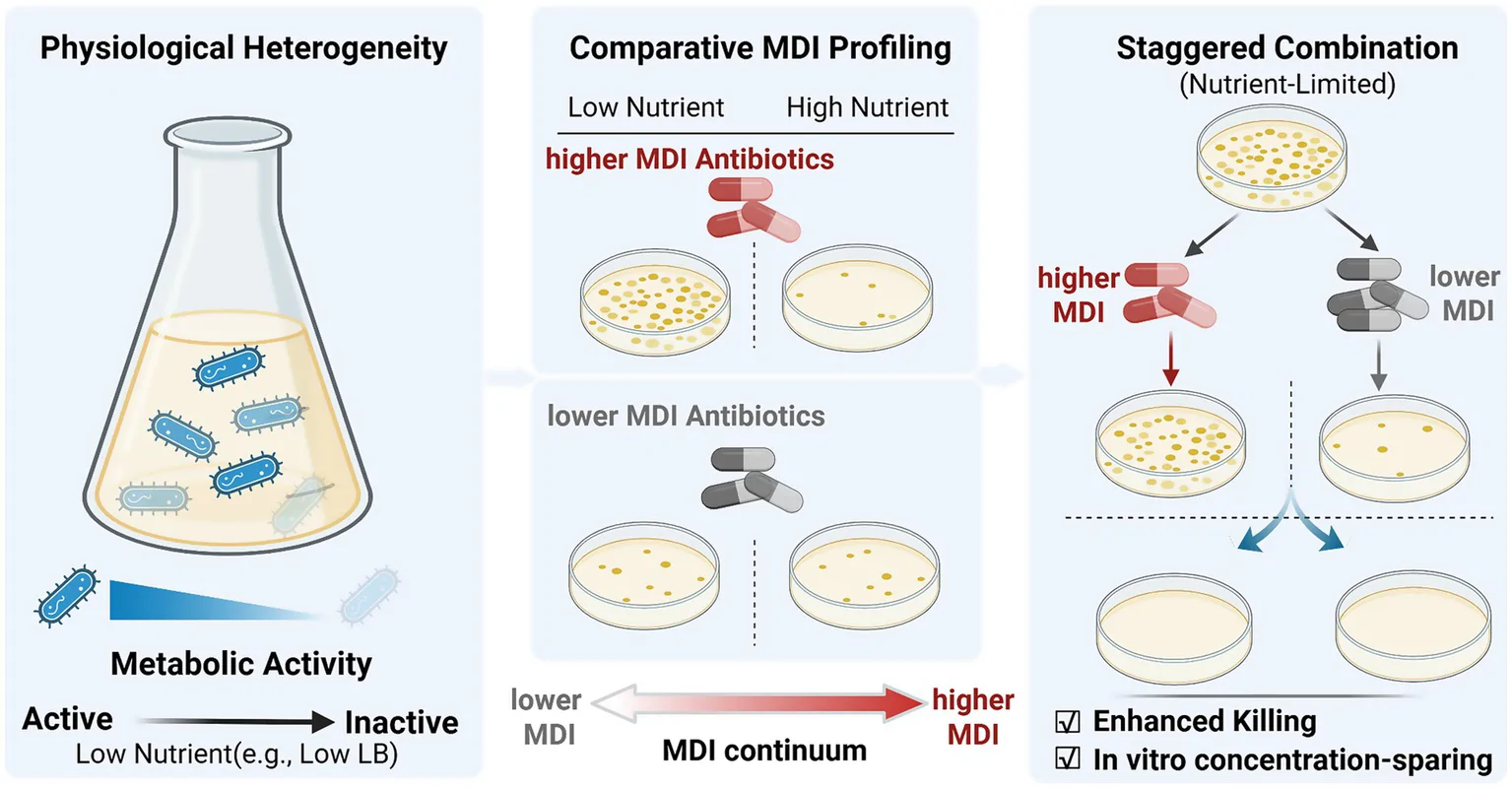
Conceptual schematic overview of a physiology-informed staggered antibiotic strategy for targeting tolerance-associated survival of *A. baumannii* under nutrient-limited conditions. Created in BioRender. Ping (2026) https://BioRender.com/9zt07ve.

## 1 Introduction

*Acinetobacter baumannii* is one of the major pathogens of hospital-acquired infections, which can lead to high-mortality infections such as ventilator-associated pneumonia, catheter-associated bloodstream infections, urinary tract infections, and secondary meningitis, particularly in critically ill patients (Yehya et al., 2025; Sharma and Lakhanpal, 2025). Owing to its ability to persist under diverse environmental stresses and rapidly acquire multidrug resistance, *A. baumannii* has been recognized as a priority pathogen for which improved therapeutic strategies are urgently needed (Scoffone et al., 2025). Although antimicrobial resistance is a major contributor to treatment failure, phenotypic antibiotic tolerance can also promote bacterial survival during antibiotic exposure and contribute to persistent or recurrent infection (Eisenreich et al., 2022). In contrast to antibiotic resistance, which involves a heritable increase in the minimum inhibitory concentration (MIC), antibiotic tolerance refers to a transient and reversible phenotype in which bacterial populations survive antibiotic exposure for prolonged periods without a corresponding increase in MIC. Such tolerance is generally associated with physiological adaptations that reduce the rate of antibiotic-mediated killing rather than with stable genetic resistance (Deventer et al., 2024; Brauner et al., 2016). Understanding how bacterial physiological states are associated with antibiotic efficacy may therefore provide a useful basis for improving treatment strategies against persistent *A. baumannii* infections.

Antibiotic tolerance can be promoted by environmental stresses, including nutrient limitation, oxidative stress, and host-associated pressures (Cotten and Davis, 2023). Under such conditions, bacteria can reprogram central metabolism and stress-response pathways, resulting in metabolically restricted or slow-growing states that reduce the efficacy of many antibiotics (Nair et al., 2026). At the population level, these stress-induced physiological changes can increase phenotypic heterogeneity and may permit subsets of cells to survive antibiotic exposure (Ciolli Mattioli, 2026). Although these survival phenotypes are generally reversible, prolonged persistence during sustained or sublethal antibiotic exposure can increase the opportunity for resistance-conferring mutations to arise and become selected, thereby facilitating the evolution of stable antibiotic resistance (Helaine et al., 2024; Boeck, 2023). Thus, physiological heterogeneity and stress-associated survival may contribute to infection persistence or relapse and may also create conditions that favor subsequent resistance evolution (Levin-Reisman et al., 2017).

Recent studies have revealed that the antimicrobial activity of many antibiotics can vary with bacterial physiological and metabolic state (Liu et al., 2020; Ahmad et al., 2025). On this basis, antibiotics have been proposed to differ in the extent to which their activity is associated with bacterial metabolic status (Zheng et al., 2020). This concept has been explored in model organisms such as *Escherichia coli* and *Klebsiella pneumoniae*, where combining antibiotics with contrasting metabolism-associated activity profiles has been reported to improve killing of tolerant bacterial populations (Seo et al., 2023). Nevertheless, whether comparable antibiotic-specific physiological-state associations occur in *A. baumannii* remains insufficiently characterized. In particular, it remains unclear whether antibiotics differ in the extent to which their activity varies across nutrient-associated physiological states, whether these differences can be quantitatively evaluated, and whether such information can be used to optimize antibiotic treatment under nutrient-limited conditions.

Therefore, this study aimed to systematically investigate how antibiotic efficacy varies across nutrient-associated physiological states in *A. baumannii*, establish a quantitative framework for comparing antibiotic-specific ATP–survival associations, and evaluate whether antibiotics with contrasting nutrient-state-associated activity profiles could be combined to improve bacterial killing under nutrient-limited conditions. By addressing these questions, we sought to provide an empirical framework for physiology-informed evaluation of antibiotic combinations and administration patterns in *A. baumannii*.

## 2 Materials and methods

### 2.1 Strains and culture conditions

The reference strain *A. baumannii* ATCC 19606ᵀ was obtained from the American Type Culture Collection (ATCC, Manassas, VA, United States) and used throughout the study. In addition, five clinical isolates were collected from sputum samples and venous catheter tips of intensive care unit (ICU) patients and identified using the VITEK^®^ 2 Compact automated identification and antimicrobial susceptibility testing system (bioMérieux, Marcy-l’Étoile, France). All strains were stored at −80 °C in Luria–Bertani broth (Solarbio, Beijing, China; Cat. No. L8291) supplemented with 20% (v/v) glycerol and routinely cultured on LB agar plates prepared in-house. For all experiments, a single colony was inoculated into 1 mL LB broth and cultured overnight at 37 °C with shaking at 200 rpm. Overnight cultures were diluted 1:100 into fresh LB broth and incubated under the same conditions until an OD_600_ of approximately 0.5 was reached, unless otherwise specified (Toh and Lin, 2024).

The five clinical isolates were de-identified before use, and no patient-identifying information was accessed. Their use was approved by the Human Research Ethics Committee of Ningbo No. 2 Hospital (approval no. PJ-NBEY-KY-2026-253-01), and the requirement for informed consent was waived.

### 2.2 Establishment of nutrient-gradient physiological states

To examine how antibiotic activity varies across nutrient-associated physiological states, cells were cultured in media containing a gradient of LB concentrations (100%, 10%, 1%, 0.1%, and 0% LB) (Lopatkin et al., 2019). Overnight cultures prepared as described above were diluted into fresh LB broth and grown to an OD_600_ of 0.1–0.2. Cells were harvested by centrifugation at 4000 × *g* for 10 min at 4 °C, washed three times with sterile phosphate-buffered saline (PBS), and resuspended in media containing the indicated nutrient concentrations (100%, 10%, 1%, 0.1%, or 0% LB). Nutrient-gradient media were prepared by proportionally reducing the components of standard LB medium while maintaining the same final volume and identical handling procedures across all experimental groups.

Bacterial suspensions were subsequently incubated at 37 °C with shaking at 200 rpm for 2 h to allow adaptation to the corresponding nutrient conditions. At the end of the adaptation period, parallel cultures maintained under identical nutrient conditions were used either for ATP quantification together with CFU enumeration or were immediately subjected to antibiotic treatment. Thus, ATP measurements used in subsequent analyses reflected the cellular energetic state at the onset of antibiotic exposure.

### 2.3 Growth kinetics across nutrient conditions

To quantify bacterial growth under the nutrient conditions used in the nutrient-limitation model, *A. baumannii* ATCC 19606^T^ was cultured in media containing 100%, 10%, 1%, 0.1%, or 0% LB. Bacterial growth was monitored by measuring OD_600_ at 20-min intervals for 24 h at 37 °C using a microplate reader. Three independent biological experiments were performed, each with three technical replicates. Technical replicates were averaged within each biological experiment before growth-rate analysis.

The maximum specific growth rate μmax (h^−1^) was estimated by linear regression of ln(OD_600_) against time according to:


$$\ln({\mathrm{OD}}_{600})=\mathrm{\mu t}+b$$

where *μ* represents the specific growth rate (h^−1^), *t* represents time (h), and *b* is the regression intercept. A four-point sliding-window linear regression, corresponding to a 1-h interval, was applied across each growth curve. Only windows showing a sustained increase in OD_600_ and a coefficient of determination R^2^ ≥ 0.95 were considered eligible. The largest positive slope among the qualifying windows was recorded as μmax for each biological replicate. For nutrient conditions in which no interval met these criteria, μmax was designated as not reliably determinable (ND).

### 2.4 Measurement of intracellular ATP levels

Intracellular ATP levels were quantified after 2 h of incubation under the indicated nutrient conditions, immediately before antibiotic exposure. ATP measurements were performed in parallel with the time-kill assays under identical nutrient conditions. Concurrently, viable bacterial counts were determined by CFU enumeration. The measured ATP concentrations were normalized to the corresponding viable cell counts and expressed as ATP per CFU, thereby accounting for differences in viable bacterial density among nutrient conditions.

Intracellular ATP levels were determined using the BacTiter-Glo™ Microbial Cell Viability Assay Kit (Promega Corporation, Madison, WI, United States; Cat. No. G8231) according to the manufacturer’s instructions. Briefly, 100 μL of bacterial suspension was mixed with an equal volume of BacTiter-Glo reagent, incubated at room temperature for 5 min, and luminescence was measured using a SpectraMax^®^ Paradigm^®^ Multi-Mode Detection Platform (Molecular Devices, San Jose, CA, United States) operated in luminescence mode. Background luminescence generated by the corresponding sterile medium was subtracted from all measurements (Mohiuddin et al., 2024). Absolute ATP concentrations were calculated using a standard curve generated from purified ATP standards (100–1,000,000 nM; 10-fold serial dilutions prepared in PBS). All ATP measurements were performed in three independent biological experiments, each with three technical replicates. Technical replicates were averaged within each biological experiment before analysis.

### 2.5 Antibiotic susceptibility determination and time-kill assays

Minimum inhibitory concentrations (MICs) were determined using the broth microdilution method according to the Clinical and Laboratory Standards Institute (CLSI) M100 guidelines (33rd edition, 2023) (Clinical and Laboratory Standards Institute, 2023). Two-fold serial dilutions of each antibiotic were prepared in 96-well microplates, and bacterial suspensions were adjusted to approximately 5 × 10^5^ CFU/mL. Plates were incubated at 37 °C for 18–20 h, and the MIC was defined as the lowest antibiotic concentration that completely inhibited visible bacterial growth (Kowalska-Krochmal and Dudek-Wicher, 2021). MIC results for meropenem, amikacin, ceftazidime, ciprofloxacin, and polymyxin B were interpreted according to CLSI breakpoints. Because CLSI does not provide susceptibility breakpoints for tigecycline against *A. baumannii*, tigecycline MICs were reported without assigning susceptibility categories, and concentrations used in subsequent assays were based on experimentally determined MICs. All antibiotics were purchased from MedChemExpress (MCE, Monmouth Junction, NJ, United States).

To evaluate antibiotic killing across nutrient-associated physiological states, time-kill assays were performed using bacterial populations prepared as described in Section 2.2 (Chung and Ko, 2019). Following the 2-h nutrient adaptation period, bacterial suspensions were adjusted to an OD_600_ of 0.1. Initial viable bacterial counts were determined by CFU enumeration immediately before antibiotic addition. Antibiotics were then added at the indicated concentrations, and cultures were incubated at 37 °C with shaking at 200 rpm for 6 h. Untreated cultures maintained under the corresponding nutrient conditions served as controls.

For all CFU enumeration, including initial viable counts and post-treatment bacterial counts, the entire 200-μL culture from each well was collected and centrifuged at 4000 × *g* for 7 min at 4 °C. The supernatant was discarded, and the bacterial pellet was resuspended in 100 μL of sterile PBS. Tenfold serial dilutions were prepared in PBS, and 10-μL aliquots of the appropriate dilutions were spotted onto LB agar plates. After overnight incubation at 37 °C, colonies were enumerated. CFU/mL in the original 200-μL culture was calculated as *N* × reciprocal dilution factor × 50, where *N* represents the number of colonies counted in a 10-μL spot. The factor of 50 accounts for both the 10-μL plating volume and the twofold concentration resulting from resuspension of the original 200-μL culture in 100 μL PBS. Under this plating scheme, one colony detected from an undiluted 10-μL spot corresponded to 50 CFU/mL in the original culture. Representative worked calculations and corresponding raw plate examples are provided in Supplementary Table S8 and Supplementary Figure S7. Samples with no colonies detected under the plating conditions used were recorded as no colonies detected (NCD) and were not assigned a numerical CFU value. Bacterial survival was calculated as the ratio of CFU/mL after 6 h of antibiotic treatment to the initial CFU/mL before treatment, and log_10_ survival was obtained by log_10_ transformation of this ratio. Based on preliminary experiments, subsequent assays under nutrient-limited conditions were performed in 0.1% LB.

To assess whether reduced antibiotic killing under nutrient limitation was associated with stable changes in antimicrobial susceptibility, *A. baumannii* ATCC 19606ᵀ was exposed to meropenem (1 μg/mL; 1 × MIC) or amikacin (4 μg/mL; 1 × MIC) in 0.1% LB for 6 h. Following antibiotic exposure, bacterial cells were washed three times with PBS to remove residual antibiotics and then cultured overnight in antibiotic-free LB. MICs were then redetermined using the same standard broth microdilution conditions described above. Three independently treated and recovered populations were evaluated for each antibiotic and compared with the parental strain.

### 2.6 RT-qPCR analysis of stress-response genes

RT-qPCR was performed to evaluate transcriptional responses associated with nutrient limitation and antibiotic exposure. Bacterial cultures were prepared under the nutrient-gradient conditions described in Section 2.2. For nutrient-comparison experiments, bacterial cells incubated in 100% LB or 0.1% LB for 6 h were collected for RNA extraction. For antibiotic-treatment experiments, cultures maintained in 0.1% LB were exposed to the indicated antibiotics for 6 h before RNA isolation.

Total RNA was extracted using the TransZol Up Plus RNA Kit (TransGen Biotech, Beijing, China; Cat. No. ER501-01-V2) according to the manufacturer’s instructions. First-strand cDNA was synthesized from total RNA using the TransScript^®^ All-in-One First-Strand cDNA Synthesis SuperMix (One-Step gDNA Removal) (TransGen Biotech, Beijing, China; Cat. No. AT341) according to the manufacturer’s protocol.

Target genes were selected to represent major stress-response pathways previously associated with bacterial stress adaptation and antibiotic responses, including toxin–antitoxin systems (*relE*, *relB*, *hth* and *gnat*), the stringent response (*relA* and *spoT*), and the DNA damage response (*recA*). Primer sequences are listed in Supplementary Table S3.

RT-qPCR was performed using TB Green^®^ Premix Ex Taq™ (Tli RNaseH Plus) (Takara Bio Inc., Shiga, Japan; Cat. No. RR420A) on an ABI 7500 Real-Time PCR System (Applied Biosystems, Foster City, CA, United States) according to the manufacturer’s instructions. Three independent biological experiments were performed, and each biological sample was analyzed in three technical replicates. Relative gene expression was calculated using the 2^^−ΔΔCt^ method, with 16S rRNA serving as the internal reference gene (Li et al., 2024).

For nutrient-comparison experiments (Supplementary Figure S3A), bacterial cultures grown in 100% LB were used as the control. For antibiotic-treatment experiments (Supplementary Figures S3B–H), untreated bacterial cultures maintained in 0.1% LB served as the control. Results are presented as fold changes relative to the corresponding control sample.

### 2.7 Calculation of the Metabolic Dependency Index (MDI)

Based on the concept that antibiotic activity may vary with bacterial physiological and metabolic states, we defined a quantitative parameter termed the Metabolic Dependency Index (MDI) (Zheng et al., 2020). The MDI was used as an empirical comparative index describing the extent to which antibiotic killing covaried with intracellular ATP levels across the nutrient-gradient conditions. Because nutrient manipulation simultaneously altered nutrient availability, bacterial growth, medium composition, and cellular energetic state, the MDI was interpreted as a nutrient-state-associated comparative index rather than as a direct measure of causal metabolic dependence.

To enable comparison among antibiotics with different MICs, all six antibiotics were evaluated at standardized relative concentrations of 0.5×, 1×, 2×, 4×, 8×, 16×, 32×, and 64× MIC. At each concentration, the corresponding log_10_ survival values measured after 6 h of antibiotic treatment were regressed against CFU-normalized ATP levels measured immediately before antibiotic exposure. The regression slope (*k*), 95% confidence interval (CI), coefficient of determination (R^2^), Pearson correlation coefficient (*r*), and *p*-value were recorded for each regression.

The MDI for each antibiotic was calculated as the arithmetic mean of the absolute values of the concentration-specific regression slopes.


$$\mathrm{MDI}=\frac{1}{n}\sum_{i=1}^{n}\;k_{i}$$

where *n* represents the number of standardized antibiotic concentrations analyzed (*n* = 8), and *kᵢ* represents the regression slope at the *i*-th concentration. Larger MDI values indicate greater ATP-associated variation in antibiotic killing across the tested nutrient states. The MDI was treated as a continuous descriptive index, and no threshold was used to classify antibiotics into discrete metabolic-dependence categories.

The ATP measurements and time-kill assays were performed using parallel cultures under identical nutrient-adaptation conditions. For each bootstrap iteration, biological replicates were resampled with replacement within each nutrient condition. Condition-specific mean CFU-normalized ATP and survival values were recalculated, followed by refitting of the concentration-specific regressions and recalculation of the MDI. This procedure was repeated 10,000 times to derive percentile-based 95% confidence intervals. Dose-specific slopes were not treated as independent biological replicates (Davison and Hinkley, 1997).

### 2.8 Evaluation of MDI-guided antibiotic combinations and administration patterns

Antibiotic combination experiments were performed under nutrient-limited conditions using *A. baumannii* ATCC 19606^T^. Bacterial cultures were prepared as described in Section 2.2 and incubated in 0.1% LB for 2 h before antibiotic exposure. Based on the continuous MDI profiles of the six antibiotics, meropenem (MEM) and amikacin (AMK), representing opposite ends of the observed MDI range, were selected for detailed evaluation in combination and staggered-administration experiments.

Three MEM–AMK administration patterns were evaluated in *A. baumannii* ATCC 19606ᵀ: simultaneous administration, MEM-first staggered administration, and AMK-first staggered administration. For simultaneous administration, MEM and AMK were added together at the beginning of the 6-h exposure period. For MEM-first staggered administration, MEM was added at 0 h, followed by AMK at 3 h without removal of MEM, and cultures were incubated for an additional 3 h. For AMK-first staggered administration, AMK was added at 0 h, followed by MEM at 3 h without removal of AMK, and incubation was continued for an additional 3 h. Thus, all three administration patterns had a total treatment duration of 6 h but differed in the timing and order of antibiotic addition (Chung and Ko, 2019).

Antibiotic combinations were evaluated using checkerboard killing assays in sterile 96-well plates. For staggered-treatment experiments, concentrations of the first-administered antibiotic were arranged along one axis and concentrations of the second-administered antibiotic along the other axis. For simultaneous treatment experiments, the same concentration matrices were used, except that both antibiotics were added at the beginning of the exposure period. Additional checkerboard killing assays were performed for MEM–polymyxin B, AMK–polymyxin B, and MEM–tigecycline combinations.

Following antibiotic exposure, bacterial cultures were processed for CFU enumeration as described in Section 2.5. Each experimental condition was evaluated in three independent biological experiments. Two prespecified MEM–AMK concentration combinations—MEM 16 μg/mL + AMK 2 μg/mL and MEM 16 μg/mL + AMK 1 μg/mL—were selected for paired comparisons between simultaneous and MEM-first staggered administration. Matched biological replicates were analyzed as described in Section 2.10. Replicate-level CFU measurements are provided in Supplementary Table S6.

### 2.9 Validation of MEM–AMK administration patterns in clinical isolates

To assess whether the killing patterns observed in the reference strain were reproducible across additional *A. baumannii* isolates, validation experiments were performed using five clinical isolates. Bacterial cultures were prepared under nutrient-limited conditions (0.1% LB), following the same procedure used for the reference strain. MEM–AMK checkerboard killing assays were performed to compare MEM-first staggered and simultaneous administration using the corresponding protocols described in Section 2.8. Following 6 h of antibiotic exposure, surviving bacterial populations were quantified by CFU enumeration as described in Section 2.5. Each experimental condition was evaluated in three independent biological experiments. Replicate-level CFU measurements are provided in Supplementary Table S7.

### 2.10 Statistical analysis

Data are presented as mean ± SEM from three independent biological experiments unless otherwise indicated. Technical replicates were averaged within each biological replicate before statistical analysis. Statistical analyses were performed using GraphPad Prism 8 (GraphPad Software, San Diego, CA, United States). Comparisons between two independent groups were performed using unpaired two-tailed Student’s *t*-tests, whereas multiple-group comparisons were analyzed using one-way ANOVA followed by Dunnett’s multiple-comparisons test (Dunnett, 1955). Prespecified comparisons between simultaneous and MEM-first staggered MEM–AMK administration were performed on log_10_-transformed CFU/mL values using paired two-tailed *t*-tests, with corresponding 95% confidence intervals reported. No NCD observations occurred in these two prespecified comparisons; all three matched biological replicates yielded quantifiable CFU/mL values. Therefore, no imputation, censoring adjustment, or pairwise exclusion was applied, and each paired test used three complete matched pairs (*n* = 3).

Linear regression was used to assess the relationship between CFU-normalized intracellular ATP levels and log_10_ bacterial survival. For the primary MDI analysis, NCD observations were assigned a pseudocount of 1 CFU/mL solely for regression-based calculation. Sensitivity analyses were performed using alternative NCD pseudocounts of 25 and 50 CFU/mL and an *R*^2^-filtered MDI calculated using only dose-specific regressions with *R*^2^ > 0.10. These sensitivity analyses were exploratory.

The MDI uncertainty was estimated by bootstrap resampling as described in Section 2.7. Dose-specific regression slopes were not treated as independent biological replicates, and no inferential comparison among antibiotics was based on these concentration-specific slopes. For heatmap presentation and prespecified paired comparisons, NCD observations were retained as categorical observations, were not interpreted as 0 CFU/mL, and were not assigned numerical CFU values. A *p*-value < 0.05 was considered statistically significant; however, given the limited number of biological replicates (*n* = 3), *p*-values from the prespecified paired comparisons were interpreted cautiously, with emphasis placed on the reported effect sizes and 95% confidence intervals.

## 3 Results

### 3.1 Nutrient limitation alters bacterial growth and cellular energetic state in *A. baumannii*

To characterize physiological changes associated with nutrient availability, *A. baumannii* ATCC 19606ᵀ was cultured across a gradient of LB concentrations ranging from 0 to 100%. The experimental design of the nutrient-gradient model is illustrated in Figure 1A. Growth kinetics differed markedly across the tested nutrient conditions (Supplementary Figure S1A). The maximum specific growth rate (μmax) decreased from 1.358 ± 0.012 h^−1^ in 100% LB to 0.831 ± 0.014 h^−1^ in 10% LB. In 1%, 0.1%, and 0% LB, no interval met the predefined criteria for reliable growth-rate estimation; therefore, μmax was designated as not reliably determinable (ND) (Supplementary Table S2), indicating marked restriction of bacterial growth under severe nutrient limitation. Consistent with these growth changes, CFU-normalized intracellular ATP levels measured after the 2-h nutrient-adaptation period were lower under nutrient-limited conditions than under nutrient-rich conditions (Figure 1B). Together, these findings indicate that the nutrient-gradient model generated distinct physiological states characterized by coordinated changes in bacterial growth and cellular energetic status, providing an experimental framework for examining nutrient-state-associated variation in antibiotic killing.

**Figure 1:**
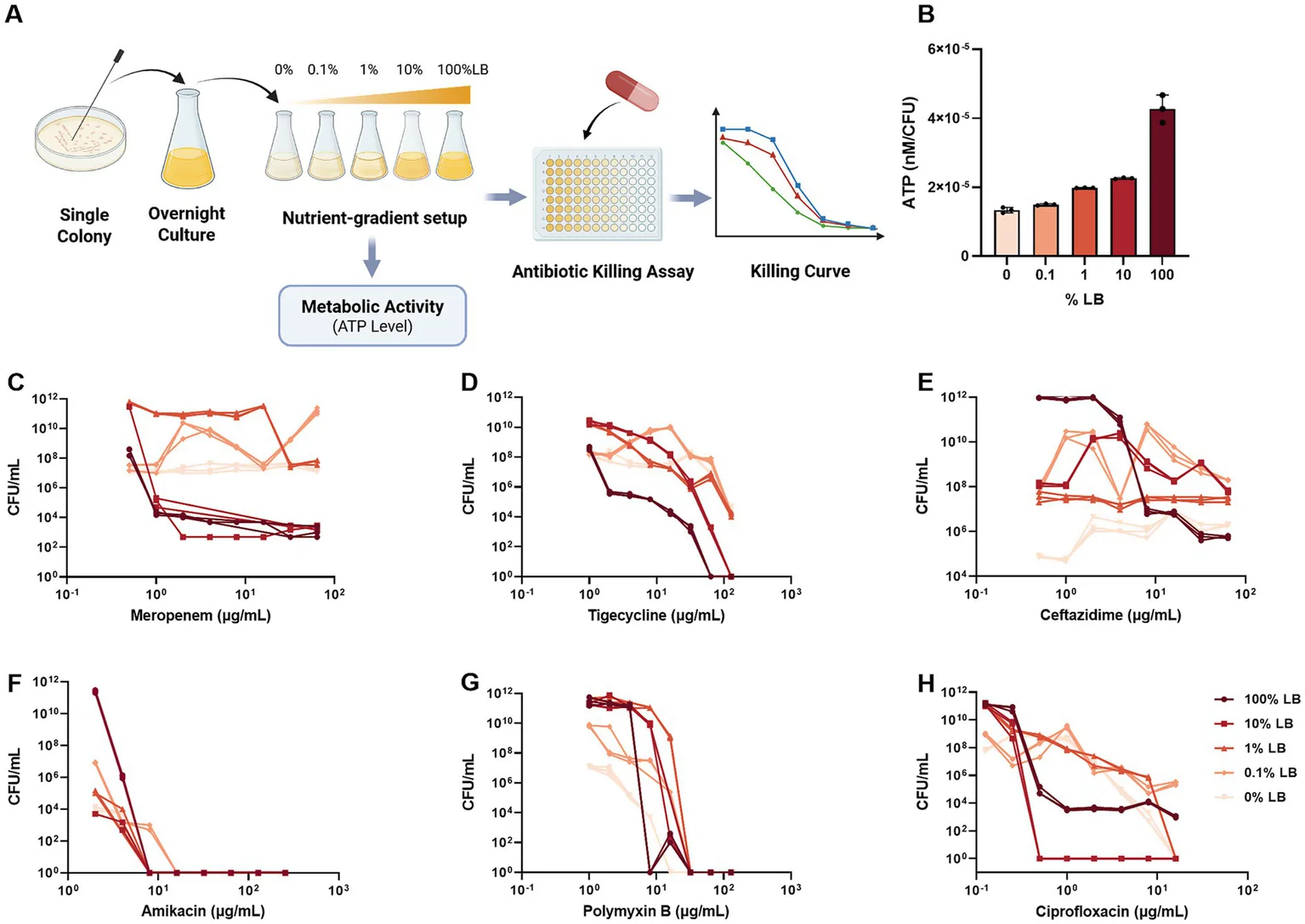
Nutrient-associated variation in antibiotic killing of *Acinetobacter baumannii ATC*C 19606^T^. **(A)** Schematic overview of the nutrient-gradient model used to generate distinct nutrient-associated physiological states. Created in BioRender. Ping (2026) https://BioRender.com/ymp07g8. **(B)** CFU-normalized ATP levels measured immediately after the 2-h nutrient-adaptation period. Individual points represent independent biological experiments, and bars indicate mean ± SEM (*n* = 3). **(C–H)** Viable bacterial counts following 6 h of antibiotic exposure across the five nutrient conditions. Antibiotics tested were **(C)** meropenem, **(D)** tigecycline, **(E)** ceftazidime, **(F)** amikacin, **(G)** polymyxin B, and **(H)** ciprofloxacin. Light-to-dark shading indicates increasing nutrient availability. Curves show mean viable bacterial counts from three independent biological experiments.

### 3.2 Antibiotic efficacy varies across nutrient-associated physiological states and is associated with intracellular ATP levels

To examine how antibiotic efficacy varies across nutrient-associated physiological states, six antibiotics were evaluated across the nutrient gradient using 6-h CFU-based killing assays. Meropenem and tigecycline showed marked reductions in killing under nutrient-limited conditions, whereas amikacin exhibited comparatively smaller changes in killing across nutrient conditions. Ceftazidime, ciprofloxacin, and polymyxin B displayed concentration-dependent changes in killing across the nutrient gradient, indicating that the observed killing patterns varied with both antibiotic identity and concentration (Figures 1C–H).

To quantify ATP–survival relationships, log_10_ survival after 6 h of antibiotic exposure was regressed against CFU-normalized intracellular ATP levels measured immediately before antibiotic addition. Separate regressions were performed across nutrient conditions for each antibiotic at each tested MIC multiple (0.5×−64× MIC). For graphical comparison, Figures 2A–F present the regressions at 1× MIC, whereas Supplementary Figure S2 displays the full concentration series. Regression slopes and correlation coefficients varied among antibiotics and across concentrations. Meropenem exhibited larger absolute regression slopes than amikacin across concentrations from 1×−64× MIC. For tigecycline, ciprofloxacin, and polymyxin B, absolute Pearson correlation coefficients were generally higher at lower or intermediate concentrations than at the highest concentrations. Most dose-specific regressions were not statistically significant (Supplementary Table S5). Consistent with these antibiotic-specific patterns, killing also differed between nutrient-rich (100% LB) and nutrient-limited (0.1% LB) conditions at 1× MIC (Figure 2G).

**Figure 2:**
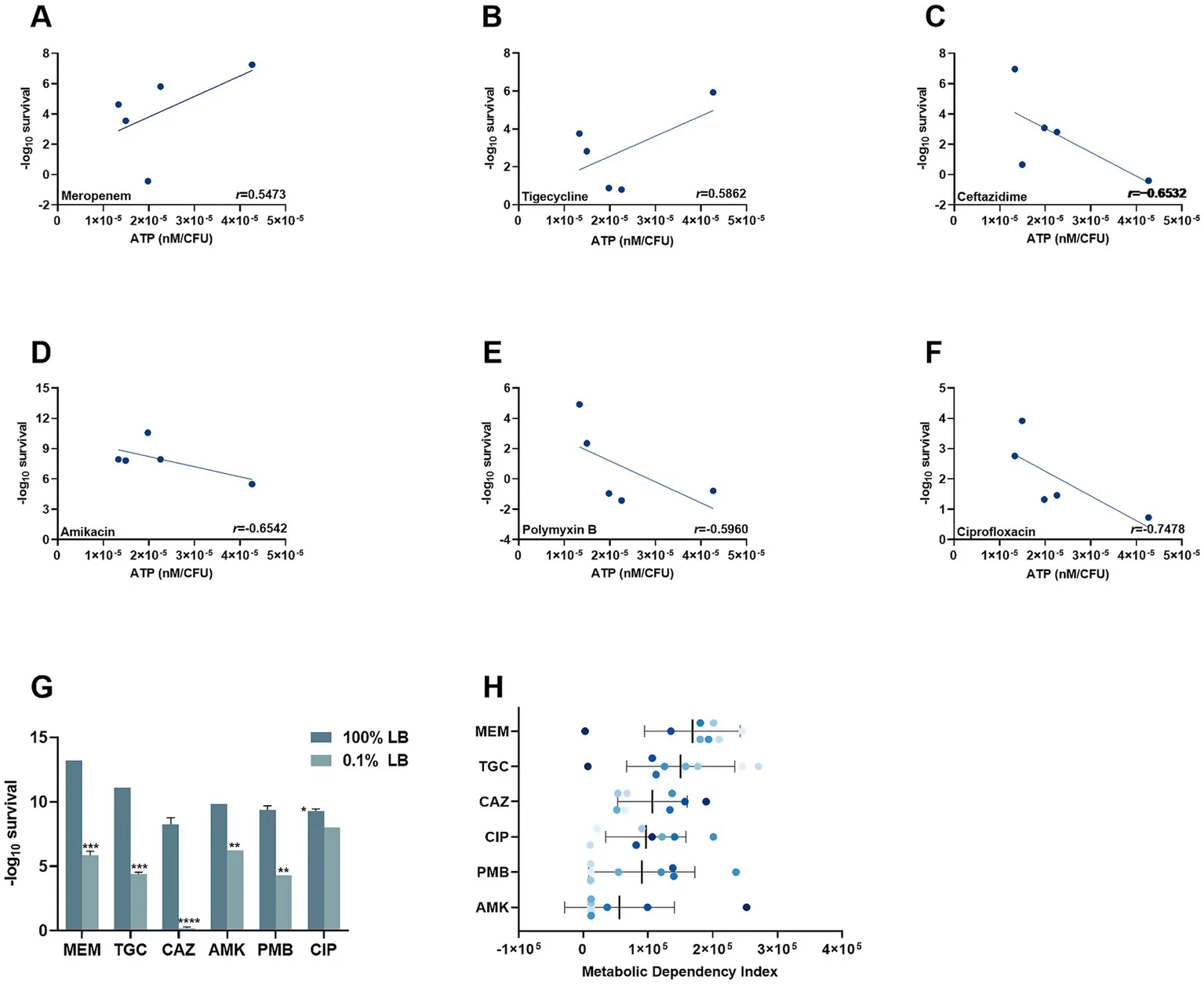
Quantitative assessment of nutrient-state-associated antibiotic responses. **(A–F)** Linear regression analyses relating CFU-normalized intracellular ATP levels measured immediately before antibiotic exposure to log_10_ bacterial survival after 6 h of treatment across nutrient conditions at 1× MIC. For graphical presentation, log_10_ survival values were multiplied by −1 and displayed as −log_10_ survival, such that higher values indicate greater bacterial killing. Consequently, the Pearson correlation coefficients (*r*) shown in these panels have signs opposite to those calculated using log_10_ survival and reported in Supplementary Table S5. Regression analyses are shown for **(A)** Meropenem, **(B)** Tigecycline, **(C)** Ceftazidime, **(D)** Amikacin, **(E)** Polymyxin B, and **(F)** Ciprofloxacin. **(G)** Comparison of -log_10_ survival under nutrient-rich (100% LB) and nutrient-limited (0.1% LB) conditions at 1× MIC. Data are presented as mean ± SEM from three independent biological replicates. Statistical comparisons between 100% LB and 0.1% LB for each antibiotic were performed using unpaired two-tailed Student’s *t*-tests. *p < 0.05, **p < 0.01, ***p < 0.001, and ****p < 0.0001. **(H)** Descriptive comparison of ATP–survival regression slope magnitudes and MDI values among the six antibiotics. Colored points represent the absolute regression slopes (|*k|*) *o*btained at the eight standardized relative concentrations from 0.5×−64× MIC. The black vertical marker indicates the MDI, calculated as the mean |*k| ac*ross the eight concentrations, and horizontal error bars indicate biological-replicate-based bootstrap 95% confidence intervals for the MDI. Dose-specific slopes were displayed descriptively and were not treated as independent biological replicates for inferential comparison among antibiotics.

To assess whether reduced killing under nutrient limitation was accompanied by a stable change in antimicrobial susceptibility, populations surviving meropenem or amikacin exposure in 0.1% LB were recovered overnight in antibiotic-free LB, after which MICs were redetermined. The meropenem MIC remained at 1 μg/mL and the amikacin MIC at 4 μg/mL in all three independently treated and recovered populations tested for each antibiotic, matching the corresponding MICs of the parental strain (Supplementary Figure S4). Thus, no increase in MIC was detected after antibiotic-free recovery, supporting a tolerance-associated interpretation of reduced killing for these two antibiotics under the tested nutrient-limited conditions.

### 3.3 Transcriptional responses associated with nutrient limitation and antibiotic exposure

To further characterize the physiological responses to nutrient limitation, transcript levels of representative stress-response genes were analyzed by RT-qPCR. Compared with bacteria cultured in 100% LB, cells maintained in 0.1% LB showed altered expression of multiple stress-associated genes (Supplementary Figure S3A). Several genes associated with toxin–antitoxin systems and the stringent response were differentially regulated under nutrient-limited conditions, whereas others showed relatively modest changes, indicating broad transcriptional remodeling.

Under nutrient-limited conditions, antibiotic exposure was associated with gene-specific transcriptional changes. The toxin–antitoxin system-related genes *hth*, *gnat*, *relE*, and *relB* were significantly upregulated following exposure to all six antibiotics, although the magnitude of induction varied among treatments (Supplementary Figures S3B–E). In contrast, the stringent-response genes *relA* and *spoT* showed relatively limited changes, except for a significant reduction in *relA* expression following ciprofloxacin treatment (Supplementary Figures S3F,G). The DNA damage-associated gene *recA* exhibited antibiotic-specific induction, with the strongest increase observed following ceftazidime exposure (Supplementary Figure S3H).

Overall, these transcriptional changes were associated with nutrient limitation and antibiotic exposure and were accompanied by coordinated stress-response remodeling. However, because only transcript abundance was measured, these data should be interpreted as associative rather than direct evidence of functional involvement in antibiotic tolerance.

### 3.4 MDI reveals differential nutrient-state associations among antibiotics

To quantitatively compare antibiotic-specific variation across the nutrient-associated physiological states, the Metabolic Dependency Index (MDI) was calculated from dose-specific linear regressions relating CFU-normalized intracellular ATP levels to subsequent log_10_ survival after 6 h of antibiotic exposure. The MDI was defined as the mean absolute regression slope (|*k*|) across the standardized concentration range of 0.5×−64× MIC and therefore reflects the extent of ATP-associated variation in antibiotic killing across the tested nutrient states.

The six antibiotics showed distinct MDI values (Figure 2H and Supplementary Table S5). Meropenem exhibited the highest MDI (168,619; bootstrap 95% CI, 147546–233,242), followed by tigecycline (150,260; 121,869–166,651), ceftazidime (106,668; 93,948–122,726), ciprofloxacin (96,631; 74,463–103,009), polymyxin B (90,142; 72,833–106,633), and amikacin (54,682; 51,576–61,452). Thus, meropenem showed the greatest ATP-associated variation in killing across the tested nutrient states, whereas amikacin showed the lowest MDI among the six antibiotics.

Sensitivity analyses using an *R*^2^-filtered slope-based formulation and alternative NCD pseudocounts produced broadly consistent antibiotic rankings (Supplementary Figure S6 and Supplementary Table S9, respectively). These analyses support the relative stability of the observed ranking, while the MDI remains an exploratory comparative index.

The MDI was treated as a continuous descriptive index rather than as a discrete mechanistic classification. Meropenem and amikacin, representing opposite ends of the observed MDI spectrum, were therefore selected for subsequent combination and administration-pattern experiments.

### 3.5 MDI-guided antibiotic combinations and administration patterns influence bacterial killing under nutrient-limited conditions

Based on the MDI analysis, meropenem and amikacin, representing opposite ends of the observed MDI spectrum, were selected to evaluate whether antibiotic combination and administration patterns could improve bacterial killing under nutrient-limited conditions. Simultaneous MEM–AMK administration enhanced killing compared with the corresponding monotherapies across multiple concentration combinations (Figure 3B). Additional checkerboard killing assays with MEM–polymyxin B, AMK–polymyxin B, and MEM–tigecycline further showed that combination-killing patterns varied according to both antibiotic pairing and concentration (Figures 3C–F).

**Figure 3:**
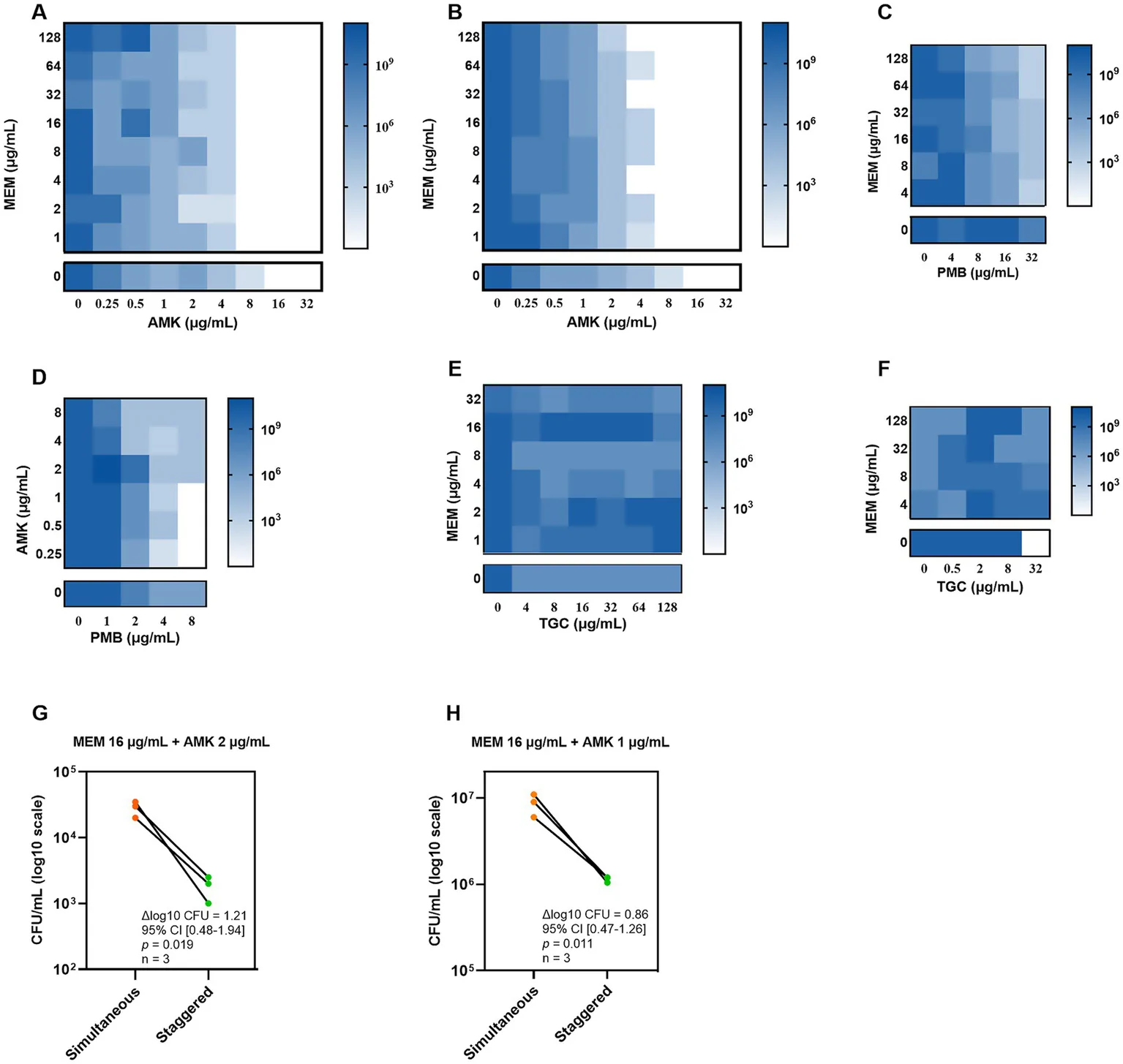
Antibiotic combination killing under nutrient-limited conditions across different administration patterns and antibiotic pairings. **(A)** MEM-first staggered MEM–AMK treatment. *A. baumannii* ATCC 19606^T^ was exposed to meropenem (MEM) for 3 h, followed by addition of amikacin (AMK) without removal of MEM, and incubation was continued for an additional 3 h. **(B)** Simultaneous MEM–AMK treatment, in which MEM and AMK were added together at the beginning of treatment and cultures were incubated for 6 h. **(C–F)** Staggered checkerboard killing assays evaluating additional antibiotic combinations under nutrient-limited conditions: **(C)** MEM–polymyxin B (PMB), **(D)** AMK–PMB, and **(E,F)** MEM–tigecycline (TGC) evaluated over two complementary concentration ranges. Heatmap colors represent mean surviving bacterial counts (CFU/mL) from three independent biological replicates and are displayed on a log_10_ scale. Darker shading indicates higher surviving bacterial counts. White cells indicate no colonies detected (NCD); NCD observations were not assigned a numerical CFU value and do not represent 0 CFU/mL. Replicate-level CFU measurements underlying the heatmaps are provided in Supplementary Table S6. **(G,H)** Replicate-level paired comparisons between simultaneous and MEM-first staggered MEM–AMK treatment at two prespecified concentration combinations: **(G)** MEM 16 μg/mL + AMK 2 μg/mL and **(H)** MEM 16 μg/mL + AMK 1 μg/mL. Each point represents one independent biological replicate, and measurements from the same biological experiment are connected. All three matched biological replicates yielded quantifiable CFU/mL values for both comparisons; no NCD observations occurred, and no imputation or pairwise exclusion was applied. Statistical comparisons were performed on log_10_-transformed CFU/mL values using paired two-tailed *t*-tests (*n* = 3 matched pairs per comparison). Treatment effects are expressed as Δlog_10_ CFU = log_10_ (CFU/mL)_simultaneous_ − log_10_ (CFU/mL)_MEM-first staggered_; positive values indicate greater bacterial killing with the MEM-first staggered regimen. Effect sizes, 95% confidence intervals, and corresponding *p*-values are shown in the figure.

We next compared simultaneous MEM–AMK administration with MEM-first staggered treatment, in which meropenem was administered for 3 h before amikacin was added without removal of meropenem. MEM-first staggered treatment resulted in lower surviving bacterial counts than simultaneous administration across multiple concentration combinations (Figures 3A,B). In two prespecified quantitative comparisons, MEM-first staggered administration reduced bacterial counts by 1.21 log_10_ CFU relative to simultaneous administration at MEM 16 μg/mL + AMK 2 μg/mL (95% CI, 0.48–1.94; paired *p* = 0.019) and by 0.86 log_10_ CFU at MEM 16 μg/mL + AMK 1 μg/mL (95% CI, 0.47–1.26; paired *p* = 0.011) (Figures 3G,H). These results indicate that the timing of MEM–AMK administration can influence bacterial killing under nutrient-limited conditions.

To determine whether this effect specifically required meropenem to precede amikacin, the reverse-order AMK-first staggered regimen was also evaluated. AMK-first staggered administration produced killing comparable to or greater than that achieved with MEM-first staggered administration under several concentration combinations (Supplementary Figure S5). Thus, the enhanced activity associated with staggered administration was not specific to a predefined MEM-first sequence, indicating that both temporal separation and antibiotic order can influence the activity of the MEM–AMK combination.

### 3.6 MEM–AMK staggered administration patterns show similar patterns across clinical isolates

To assess whether the MEM–AMK killing patterns observed in the reference strain were also detectable across additional *A. baumannii* isolates, five clinical isolates were evaluated under the same nutrient-limited condition (0.1% LB). Both simultaneous and MEM-first staggered MEM–AMK administration produced substantial bacterial killing across the tested isolates, although the magnitude of the response varied among strains (Figure 4 and Supplementary Table S7). At several concentration combinations, MEM-first staggered administration resulted in lower surviving bacterial counts than simultaneous treatment, indicating that the administration pattern influenced MEM–AMK activity across multiple clinical isolates.

**Figure 4:**
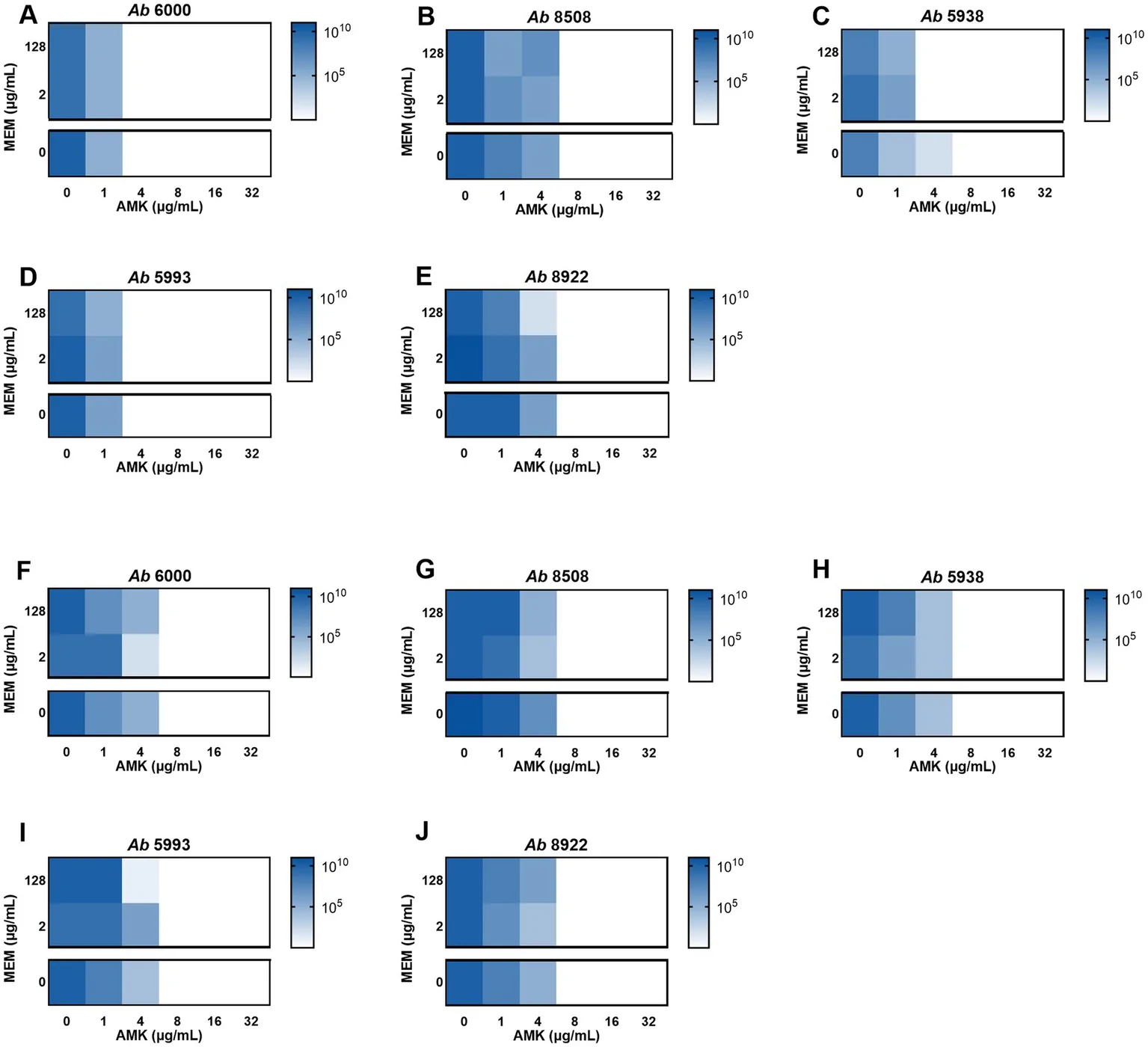
MEM–AMK administration patterns across five clinical *A. baumannii* isolates. **(A–E)** MEM-first staggered administration under nutrient-limited conditions (0.1% LB), in which meropenem (MEM) was added at 0 h followed by amikacin (AMK) at 3 h without removal of MEM. **(F–J)** Simultaneous MEM–AMK administration under the same nutrient-limited conditions, in which MEM and AMK were added together at the beginning of the 6-h exposure period. Within each treatment set, panels correspond to clinical isolates 6,000 **(A,F)**, 8,508 **(B,G)**, 5,938 **(C,H)**, 5,993 **(D,I)**, and 8,922 **(E,J)**. Heatmaps show mean surviving bacterial counts (CFU/mL) from three independent biological experiments. Darker shading indicates higher surviving bacterial counts. White cells indicate no colonies detected (NCD); NCD observations were not assigned a numerical CFU value and do not represent 0 CFU/mL. Replicate-level CFU measurements are provided in Supplementary Table S7.

Notably, enhanced killing was observed with selected combinations containing lower amikacin concentrations than those required for comparable killing with amikacin monotherapy, suggesting an *in vitro* amikacin concentration-sparing effect under the tested conditions. Overall, differences between simultaneous and MEM-first staggered administration observed in the reference strain were also detected across the five clinical isolates, although the magnitude of these differences varied among isolates.

## 4 Discussion

Antibiotic tolerance has emerged as a major challenge in the treatment of persistent bacterial infections, particularly those caused by opportunistic pathogens such as *A. baumannii* (Sulaiman and Lam, 2021). Unlike genetically encoded antibiotic resistance, antibiotic tolerance represents a reversible physiological state in which a fraction of a bacterial population survives antibiotic exposure without acquiring stable heritable resistance mutations. Increasing evidence indicates that bacterial metabolic state is an important factor influencing antibiotic tolerance (Westblade et al., 2020); however, practical approaches for quantitatively incorporating metabolic information into antibiotic evaluation and treatment design remain limited (Acierno et al., 2025; Stokes et al., 2019). In this study, we examined how antibiotic efficacy varied across nutrient-associated physiological states in *A. baumannii* and whether these differences could inform the evaluation of antibiotic combination and administration patterns. Nutrient limitation was accompanied by coordinated changes in bacterial growth and cellular energetic status and was associated with reduced killing by several clinically relevant antibiotics. The extent of ATP-associated variation in antibiotic killing differed among antibiotics and across concentrations. On this basis, we developed the Metabolic Dependency Index (MDI) as a continuous empirical framework for comparing antibiotic-specific ATP–survival associations across nutrient states. Using meropenem and amikacin, which represented opposite ends of the observed MDI spectrum, we further showed that both antibiotic combination and administration pattern influenced bacterial killing under nutrient-limited conditions. Together, these findings support considering bacterial physiological state as a quantitative variable in the evaluation and optimization of antibiotic combinations under nutrient-limited conditions.

Our results indicate that antibiotic killing varied substantially across nutrient-associated physiological states, but the magnitude of this variation differed among antibiotics and across concentrations. Meropenem showed a marked reduction in killing under nutrient-limited conditions and exhibited greater ATP-associated variation across the tested nutrient states. This observation is consistent with previous studies demonstrating that the activity of antibiotics targeting growth-associated cellular processes can be strongly affected by bacterial physiological state (Stokes et al., 2019; Peng et al., 2025; Lobritz et al., 2015). In contrast, amikacin showed comparatively smaller changes in killing across the nutrient gradient. One possible explanation is that aminoglycoside uptake relies in part on membrane potential and proton motive force, and residual membrane potential may persist even when overall metabolic activity is reduced (Wan et al., 2024; Webster and Shepherd, 2022). In addition, aminoglycosides have been reported to enhance the target-site penetration of imipenem in *A. baumannii*, suggesting that permeability-related interactions may contribute to the enhanced activity of carbapenem–aminoglycoside combinations (Yadav et al., 2015). Because membrane permeability and carbapenem target-site penetration were not directly measured in the present study, this explanation should be regarded as a possible contributing factor rather than definitive evidence. Recent evidence also indicates that polymyxin B lethality at clinically relevant concentrations requires metabolic activity, involving energy-dependent outer membrane disruption and LPS loss (Borrelli et al., 2025). This finding is consistent with our observation that polymyxin B exhibited context-dependent ATP-associated killing patterns and should not be regarded as a purely metabolism-independent antibiotic. Collectively, these findings indicate that bacterial physiological state influences antibiotic efficacy in a heterogeneous rather than uniform manner, providing a biological rationale for quantitatively comparing antibiotic-specific physiological-state associations.

The transcriptional analyses provide additional physiological context for the changes observed under nutrient-limited conditions. Rather than serving as direct mechanistic evidence, the RT-qPCR results indicate that nutrient limitation and antibiotic exposure were accompanied by gene-specific changes in the expression of selected stress-associated genes. These transcriptional responses complement the growth and ATP measurements, suggesting that the nutrient-associated physiological states examined here involved broader stress adaptation in addition to changes in cellular energetic status. Among the genes examined, components of the toxin–antitoxin system, including *relE* and *relB*, as well as the DNA damage-associated gene *recA* displayed gene-specific expression changes following nutrient limitation and antibiotic exposure. Previous studies have implicated toxin–antitoxin systems in bacterial stress adaptation and growth regulation, whereas *recA* is involved in bacterial responses to DNA damage and antibiotic-associated stress (Alkasir et al., 2018; Kashyap et al., 2021). Nevertheless, because transcript abundance was assessed at a single time point without functional genetic validation, these observations should be interpreted as supportive associations rather than as evidence of causal involvement in antibiotic tolerance.

An important contribution of the present study is the establishment of a quantitative framework for comparing antibiotic-specific associations with nutrient-associated physiological states. Previous studies have demonstrated that bacterial physiological and metabolic states can influence antibiotic activity, but practical approaches for quantitatively comparing these relationships across clinically relevant antibiotics under standardized conditions remain limited (Stokes et al., 2019; Zheng et al., 2024). To address this gap, we developed the Metabolic Dependency Index (MDI), an empirical metric derived from the relationship between CFU-normalized intracellular ATP levels and subsequent log_10_ survival across multiple standardized antibiotic concentrations. Rather than representing an absolute biological parameter or a direct measure of causal metabolic dependence, the MDI provides a continuous comparative index describing the extent of ATP-associated variation in antibiotic killing within the same experimental context. The distinct MDI profiles observed among the six antibiotics further indicate that these physiological-state associations are drug- and concentration-dependent rather than adequately captured by a binary classification. Thus, the principal value of the MDI lies in providing a standardized quantitative framework for comparing antibiotic responses across nutrient-associated physiological states and for informing subsequent antibiotic combination and administration-pattern experiments.

Beyond the antibiotic-specific differences observed across nutrient-associated physiological states, our findings further show that the pattern of MEM–AMK administration can influence combination activity. Under nutrient-limited conditions, MEM-first staggered administration produced greater killing than simultaneous administration at several concentration combinations, including the two prespecified comparisons. Interestingly, reversing the order of administration did not abolish this effect, as AMK-first staggered administration produced killing comparable to or greater than that achieved with MEM-first staggered administration under several conditions. These observations suggest that the enhanced activity associated with staggered administration may reflect dynamic interactions arising from temporal separation of antibiotic addition rather than a fixed requirement for meropenem to precede amikacin. Such interactions could involve antibiotic-induced changes in bacterial physiology, membrane properties, or drug accessibility that alter the response following addition of the second agent. Mechanism-based modeling has suggested that aminoglycosides may enhance the target-site penetration of imipenem in *A. baumannii*, providing one possible explanation for the enhanced activity of carbapenem–aminoglycoside combinations. More broadly, the present findings indicate that temporal separation of antibiotic administration can alter combination activity, although the underlying cellular mechanisms remain to be defined.

From a translational perspective, physiology-informed antibiotic strategies may be relevant to difficult-to-eradicate *A. baumannii* infections. In many infection-associated environments, including biofilms, necrotic tissues, and poorly vascularized environments, bacteria frequently experience nutrient limitation and other stresses that promote slow-growing or energetically restricted physiological states. Such conditions can reduce antibiotic killing and favor bacterial survival during treatment, thereby contributing to persistence, relapse, and potentially promoting resistance evolution (Helaine et al., 2024). Importantly, the differences between simultaneous and staggered MEM–AMK administration observed in the reference strain were also detected across five clinical isolates, although the magnitude of these differences varied among strains. In addition, selected MEM–AMK combinations achieved enhanced killing at lower amikacin concentrations than amikacin monotherapy, indicating an *in vitro* amikacin concentration-sparing effect under nutrient-limited conditions. These findings suggest that considering bacterial physiological state together with antibiotic combination and administration pattern may provide a useful framework for evaluating and optimizing antibacterial activity in physiologically constrained environments.

Despite these findings, several limitations should be acknowledged. First, the present study used an *in vitro* nutrient-limitation model to generate distinct physiological states associated with altered antibiotic killing. Nutrient manipulation simultaneously changed bacterial growth, medium composition, and cellular energetic status, making it difficult to isolate the contribution of metabolism from other nutrient-associated physiological changes. In addition, CFU-normalized intracellular ATP levels were measured immediately before antibiotic exposure and therefore represent the pre-antibiotic energetic state rather than dynamic physiological changes during the subsequent 6-h treatment period. Second, an additional methodological limitation of the MDI framework concerns the variable statistical strength of the concentration-specific regressions from which it is calculated. Several dose-specific ATP–survival regressions exhibited low coefficients of determination (R^2^) and did not reach formal statistical significance, particularly at higher antibiotic concentrations where bactericidal activity approached saturation. This pattern is biologically consistent with a concentration-dependent weakening of the influence of pre-treatment physiological state on antibiotic efficacy. Nevertheless, because the MDI is derived by averaging absolute regression slopes across all eight standardized concentrations, it represents a broad, cross-concentration descriptive index rather than a metric supported by statistically significant associations at every individual dose. The MDI therefore best serves as a comparative tool for ranking overall nutrient-state responsiveness across antibiotics, and caution should be applied when interpreting its value at any single concentration or extrapolating it to clinical dosing scenarios. Third, the MEM–AMK administration experiments were performed in a limited number of clinical isolates, all of which were carbapenem susceptible. Although differences between simultaneous and staggered administration were also observed in these isolates, the applicability of these findings to carbapenem-resistant *A. baumannii* (CRAB) remains unknown. Moreover, the reverse-order experiments showed that AMK-first staggered administration could produce killing comparable to or greater than MEM-first treatment under several conditions, indicating that the observed benefit of staggered administration is not explained by a uniquely favorable MEM-first sequence. Broader validation across genetically and phenotypically diverse clinical isolates, together with animal infection models and clinically relevant pharmacokinetic–pharmacodynamic studies, will be required to determine the generalizability of these findings. Finally, the molecular mechanisms underlying the observed nutrient-state-associated and administration-pattern-dependent differences in antibiotic killing remain incompletely defined. The transcriptional data provide evidence of stress-response remodeling but do not establish causal regulatory pathways, and the roles of membrane potential, antibiotic uptake, and dynamic physiological transitions were not directly measured. In addition, the recovery experiments addressed short-term phenotypic stability but did not assess genetic adaptation during prolonged or repeated antibiotic exposure. Future investigations integrating functional genetics with transcriptomic, metabolomic, and genomic approaches will be important for elucidating how metabolic adaptation influences antibiotic tolerance and treatment responses in *A. baumannii*.

In conclusion, this study shows that antibiotic killing of *A. baumannii* varies across nutrient-associated physiological states and that the extent of ATP-associated variation differs among antibiotics and across concentrations. The MDI provides a continuous empirical framework for quantitatively comparing these nutrient-state-associated differences under standardized experimental conditions. The MEM–AMK combination and staggered administration enhanced bacterial killing under nutrient-limited conditions, while reverse-order experiments showed that this effect was not unique to a predefined MEM-first sequence. Together, these findings provide a quantitative framework for integrating bacterial physiological state into the evaluation and optimization of antibiotic combinations under nutrient-limited conditions (Figure 5).

**Figure 5:**
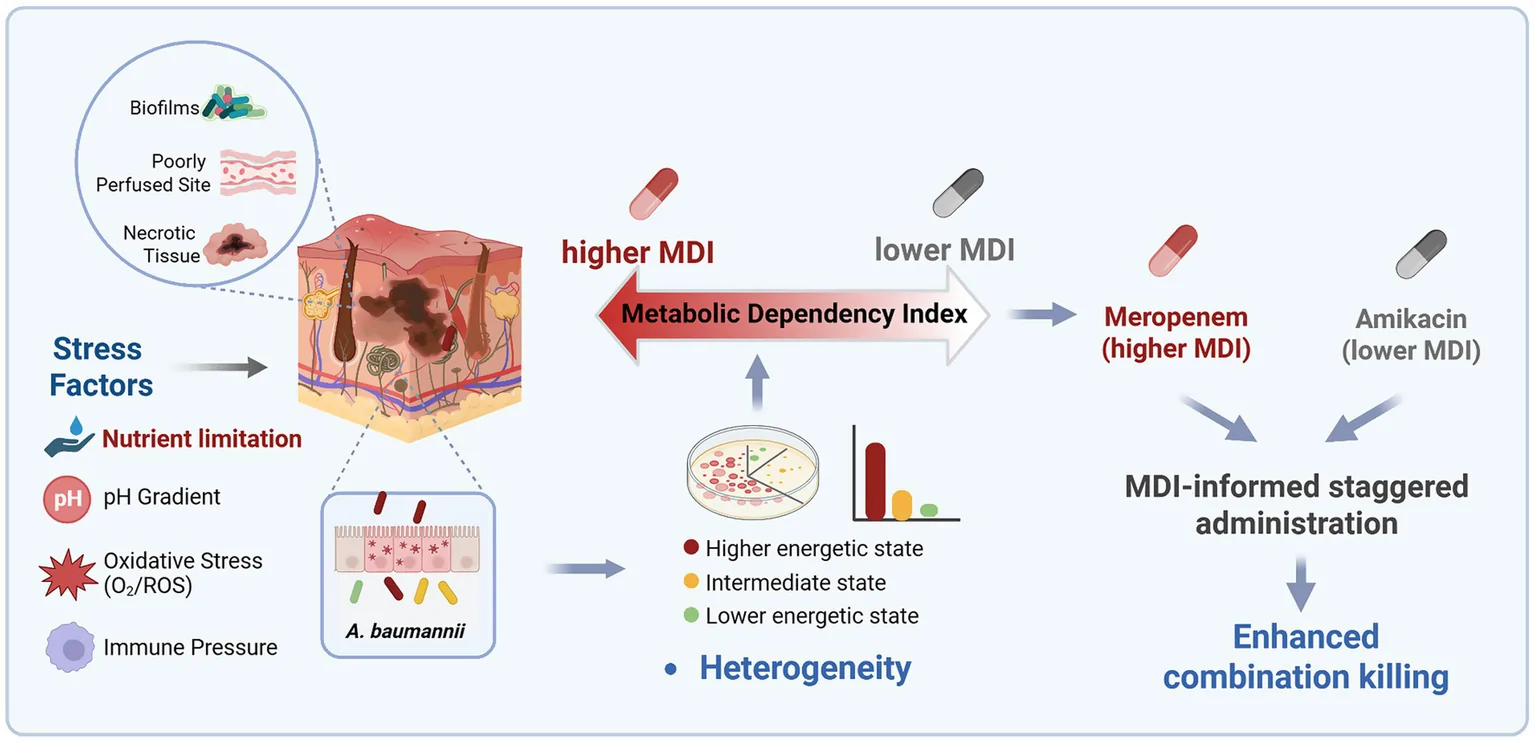
Conceptual framework for physiology-informed antibiotic combination and staggered administration in *A. baumannii.* This schematic summarizes potential future directions for the development of physiology-informed antibiotic strategies against *A. baumannii*, including model refinement, mechanistic studies of nutrient-associated physiological heterogeneity, and infection-context-guided therapeutic applications. Created in BioRender. Ping (2026) https://BioRender.com/knp4hxc.

## Data Availability

The original contributions presented in the study are included in the article/Supplementary material, further inquiries can be directed to the corresponding authors.
