# Supplementary material for: Metabolic dependency profiling guides staggered antibiotic administration to enhance killing of antibiotic-tolerant Acinetobacter baumannii
Source: Front Microbiol. 2026 Oct 29;17:1853821. doi: 10.3389/fmicb.2026.1853821 (PMC13755201; doi:10.3389/fmicb.2026.1853821)
Supplement: Supplementary file 1 [file Data_Sheet_1.PDF]

Supplementary Material

1 Supplementary Figures

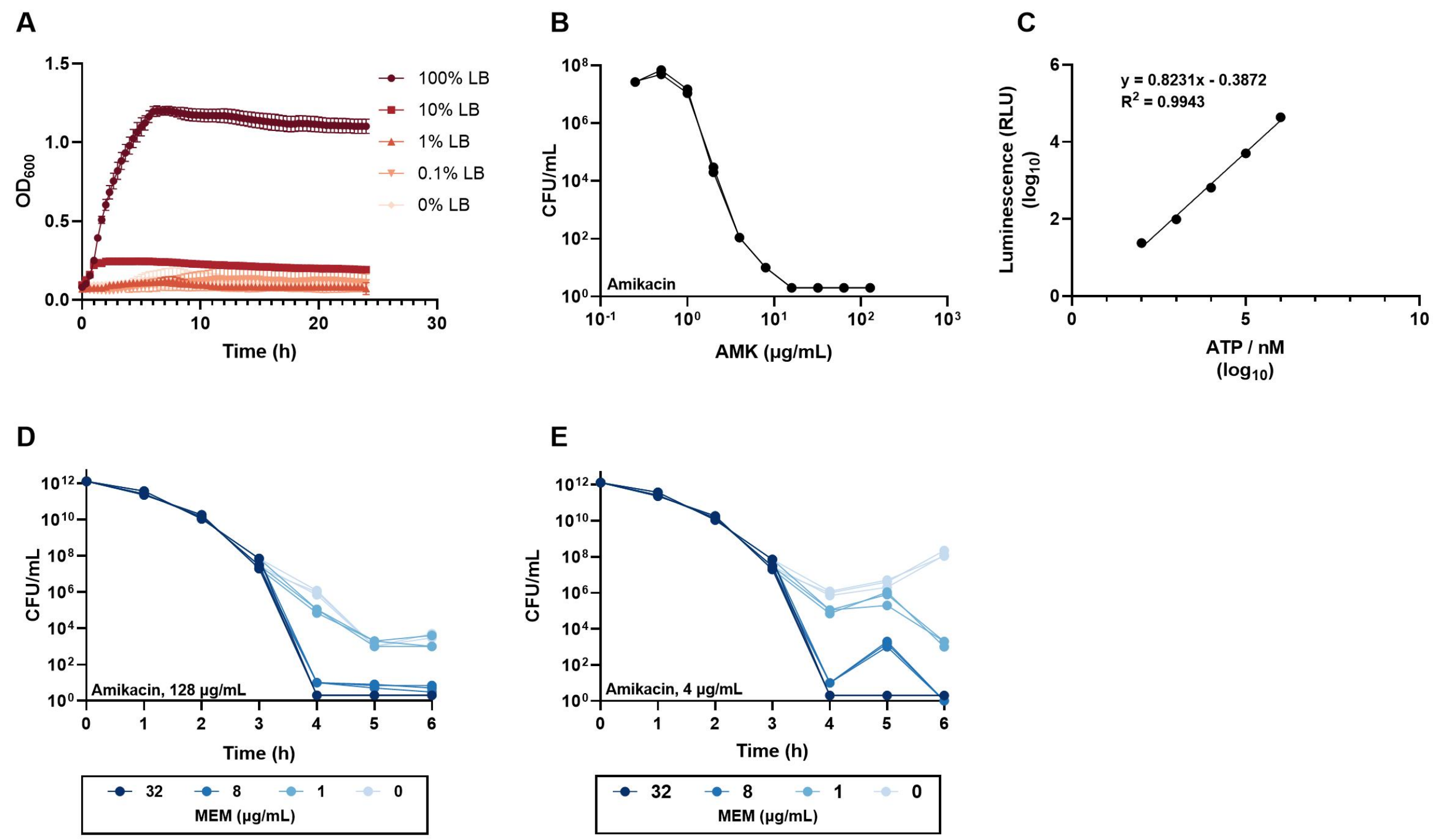

Supplementary Figure S1. Growth kinetics, amikacin monotherapy killing, ATP calibration, and MEM-first staggered treatment kinetics.

(A) Growth kinetics of *A. baumannii* ATCC 19606^T under different nutrient conditions. Bacterial cultures were incubated in media containing 100%, 10%, 1%, 0.1%, or 0% LB, and OD_{600} was monitored at 20-min intervals for 24 h. Data are presented as mean ± SEM from three independent biological experiments, each with three technical replicates. **(B)** Dose-dependent killing by amikacin after 6 h under nutrient-limited conditions (0.1% LB). **(C)** Standard calibration curve used for intracellular ATP quantification. Luminescence and ATP concentrations are shown on $\log_{10}$-transformed scales. **(D–E)** Time-kill kinetics of MEM-first staggered treatment at fixed amikacin concentrations. *A. baumannii* cultures were exposed to meropenem for 3 h, followed by addition of amikacin at 128 µg/mL **(D)** or 4 µg/mL **(E)** without removal of meropenem, followed by an additional 3 h of incubation. Data are from three independent biological experiments.

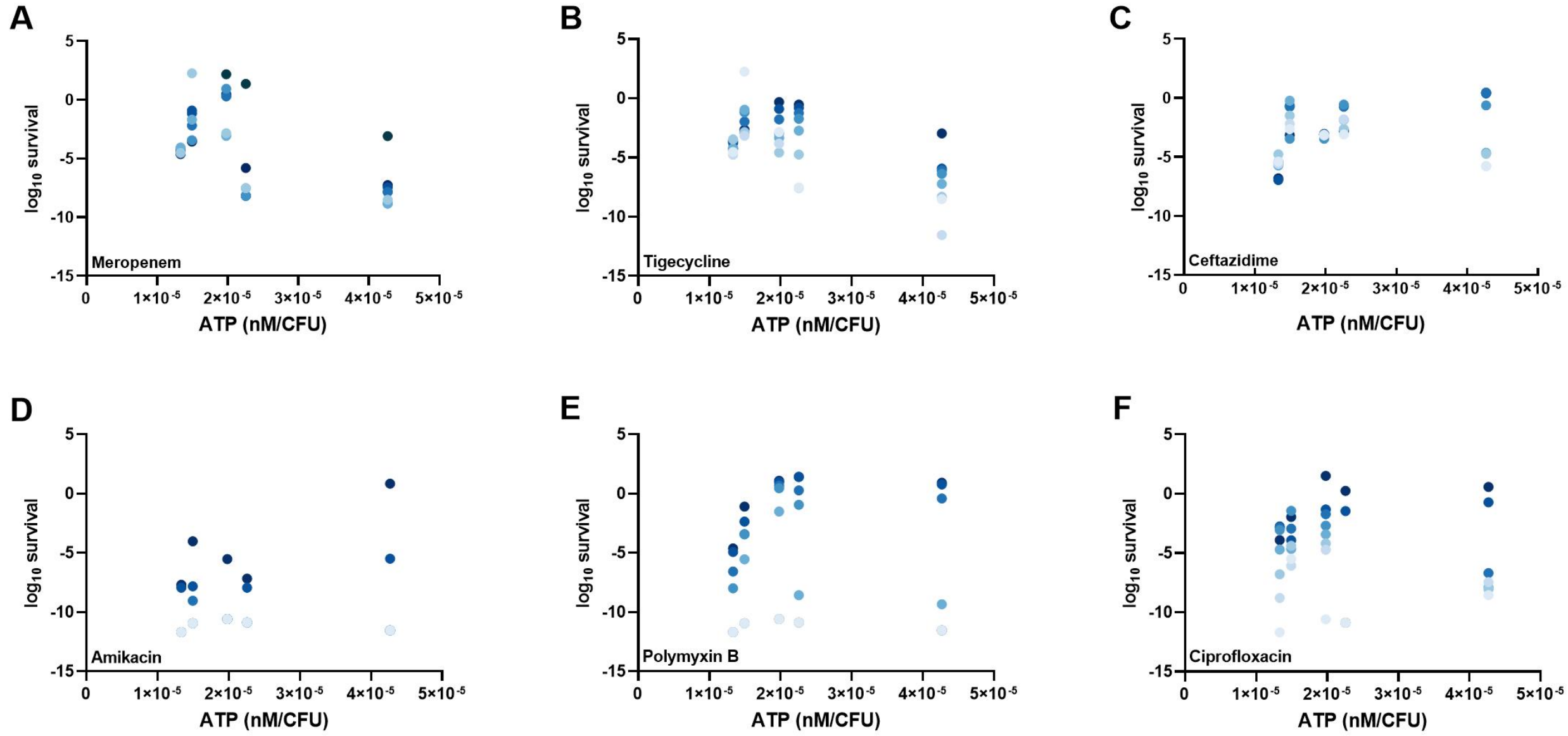

Supplementary Figure S2. Relationship between pre-antibiotic CFU-normalized intracellular ATP levels and subsequent bacterial survival following antibiotic exposure.

(**A**–**F**) Relationships between CFU-normalized intracellular ATP levels (nM/CFU) measured after the 2-h nutrient adaptation period, immediately before antibiotic addition, and subsequent $\log_{10}$ bacterial survival determined after 6 h of antibiotic exposure. All standardized antibiotic concentrations from 0.5×–64× MIC were included in both the plotted data and the MDI calculation. Light-to-dark shading indicates increasing antibiotic concentration. **(A)** Meropenem; **(B)** tigecycline; **(C)** ceftazidime; **(D)** amikacin; **(E)** polymyxin B; **(F)** ciprofloxacin.

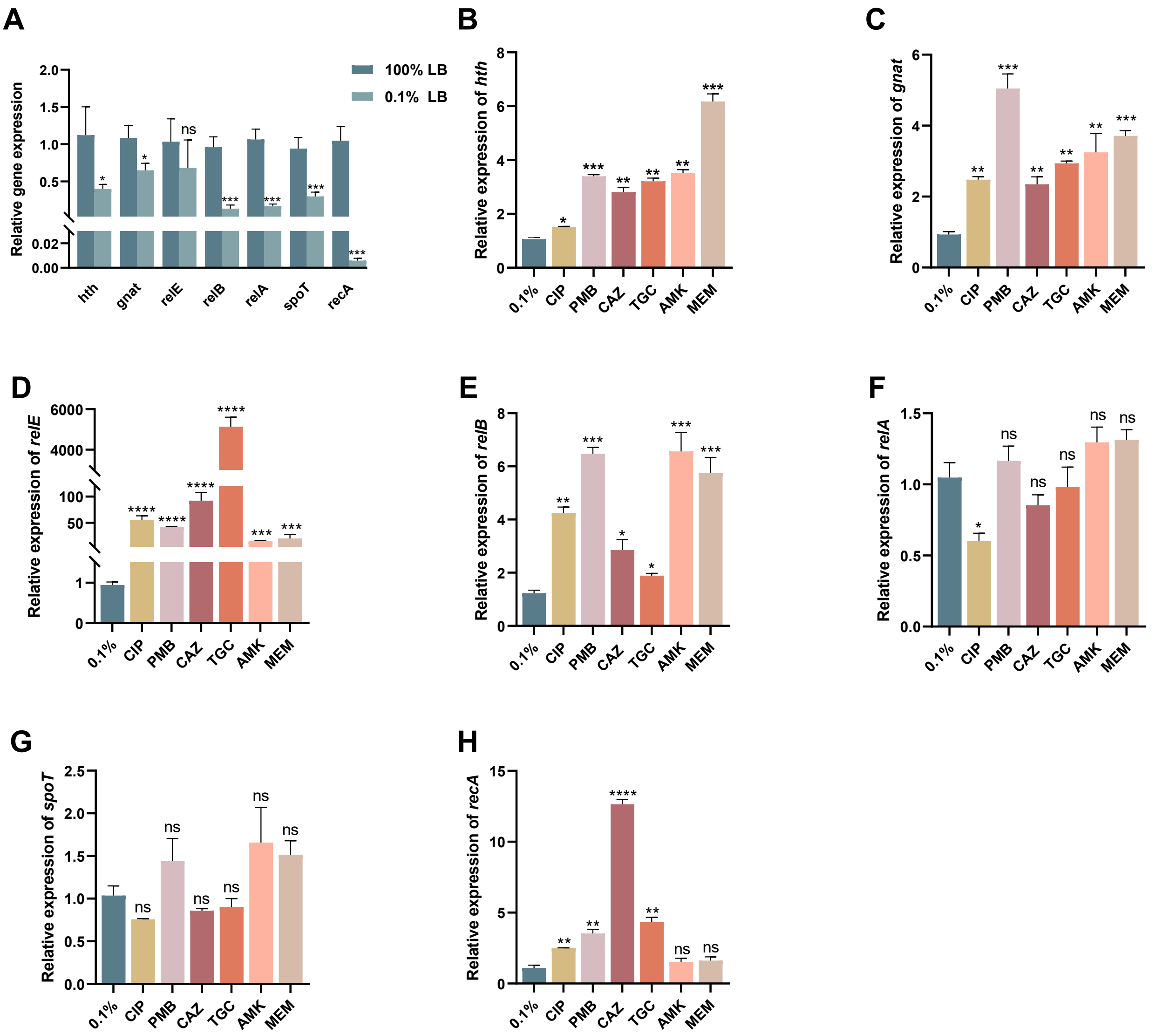

Supplementary Figure S3. Gene expression profiles associated with nutrient limitation and antibiotic exposure.

(A) Relative expression of selected toxin–antitoxin (TA) system genes, (p)ppGpp-related genes, and DNA damage-associated genes under nutrient-rich (100% LB) and nutrient-limited (0.1% LB) conditions. **(B–H)** Relative expression of selected stress-related genes under nutrient-limited conditions following antibiotic exposure. Genes analyzed included TA system genes (*relE*, *relB*, *hth*, and *gnat*), (p)ppGpp-related genes (*relA* and *spoT*), and the DNA damage-associated gene *recA*. Relative expression levels were calculated using the $2^{\wedge-\Delta\Delta Ct}$ method. For panel A, gene expression in bacteria cultured in 100% LB was used as the calibrator. For panels B–H, gene expression in bacteria cultured in 0.1% LB without antibiotic treatment was used as the calibrator. Data are presented as mean ± SEM from three independent biological replicates. ns, not significant; **p* < 0.05, ***p* < 0.01, ****p* < 0.001, *****p* < 0.0001.

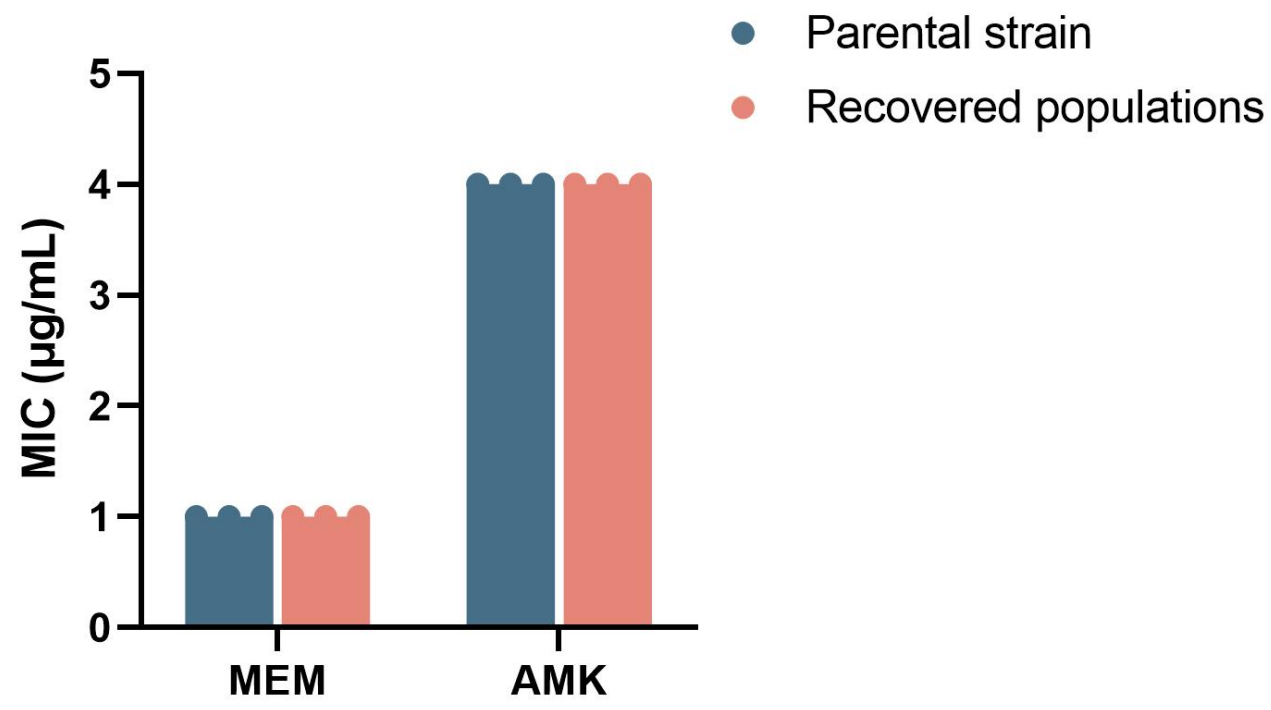

Supplementary Figure S4. MICs of parental and recovered *A. baumannii* populations following antibiotic exposure under nutrient-limited conditions.

Surviving populations of *A. baumannii* ATCC 19606^{T} following 6 h of exposure to meropenem (MEM; 1 µg/mL) or amikacin (AMK; 4 µg/mL) in 0.1% LB were recovered overnight in antibiotic-free medium, and MICs were subsequently determined using the standard broth microdilution method. For each antibiotic, three independently treated and recovered populations were evaluated. The meropenem MIC remained at 1 µg/mL and the amikacin MIC remained at 4 µg/mL in all recovered populations, identical to the parental strain. Three independent MIC determinations of the parental strain were performed for comparison. Each point represents an independent determination.

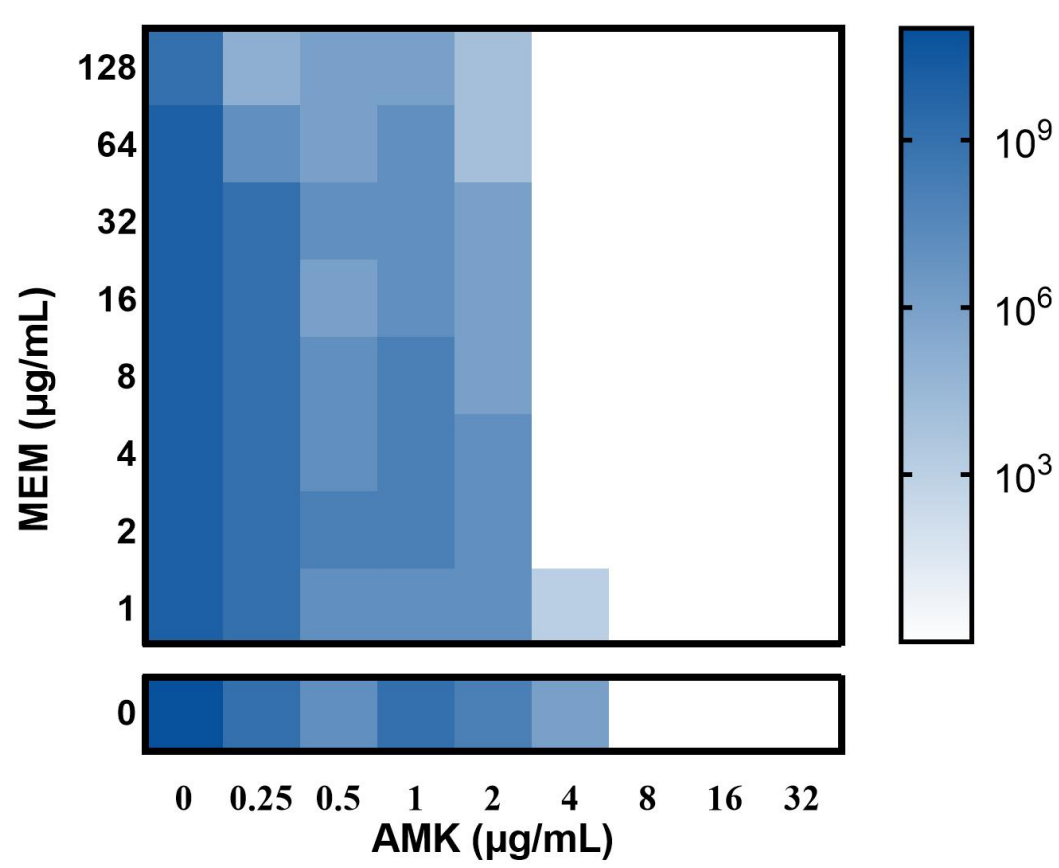

Supplementary Figure S5. Reverse-order AMK-first staggered MEM–AMK treatment under nutrient-limited conditions.

Amikacin (AMK) was added at 0 h, followed by meropenem (MEM) at 3 h without removal of AMK, and incubation was continued for an additional 3 h. The heatmap shows mean surviving bacterial counts (CFU/mL) from three independent biological experiments following AMK-first staggered treatment under nutrient-limited conditions (0.1% LB). Darker shading indicates higher surviving bacterial counts. White cells indicate no colonies detected (NCD); NCD observations were not assigned a numerical CFU value and do not represent 0 CFU/mL. Replicate-level CFU counts are provided in Supplementary Table S6g.

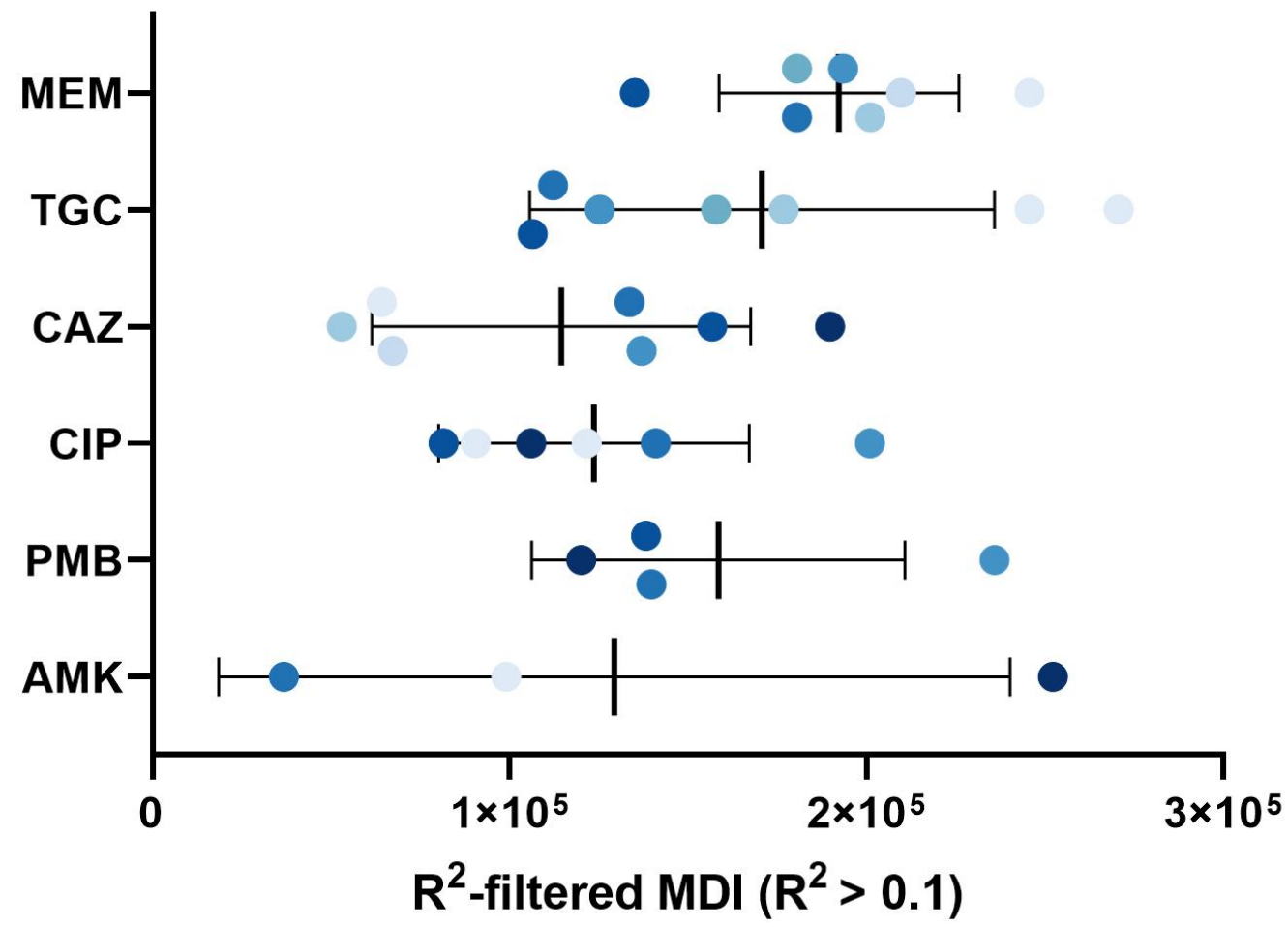

Supplementary Figure S6. R^2-filtered sensitivity analysis of the Metabolic Dependency Index. The R^2-filtered MDI was calculated as the mean absolute regression slope among dose-specific regressions with $R^2 > 0.10$. Colored points represent dose-specific absolute regression slopes ($|k|$). Black vertical markers indicate the corresponding mean index values. Horizontal error bars represent the standard deviation across dose-specific values and are shown for descriptive purposes only; they do not represent biological-replicate uncertainty.

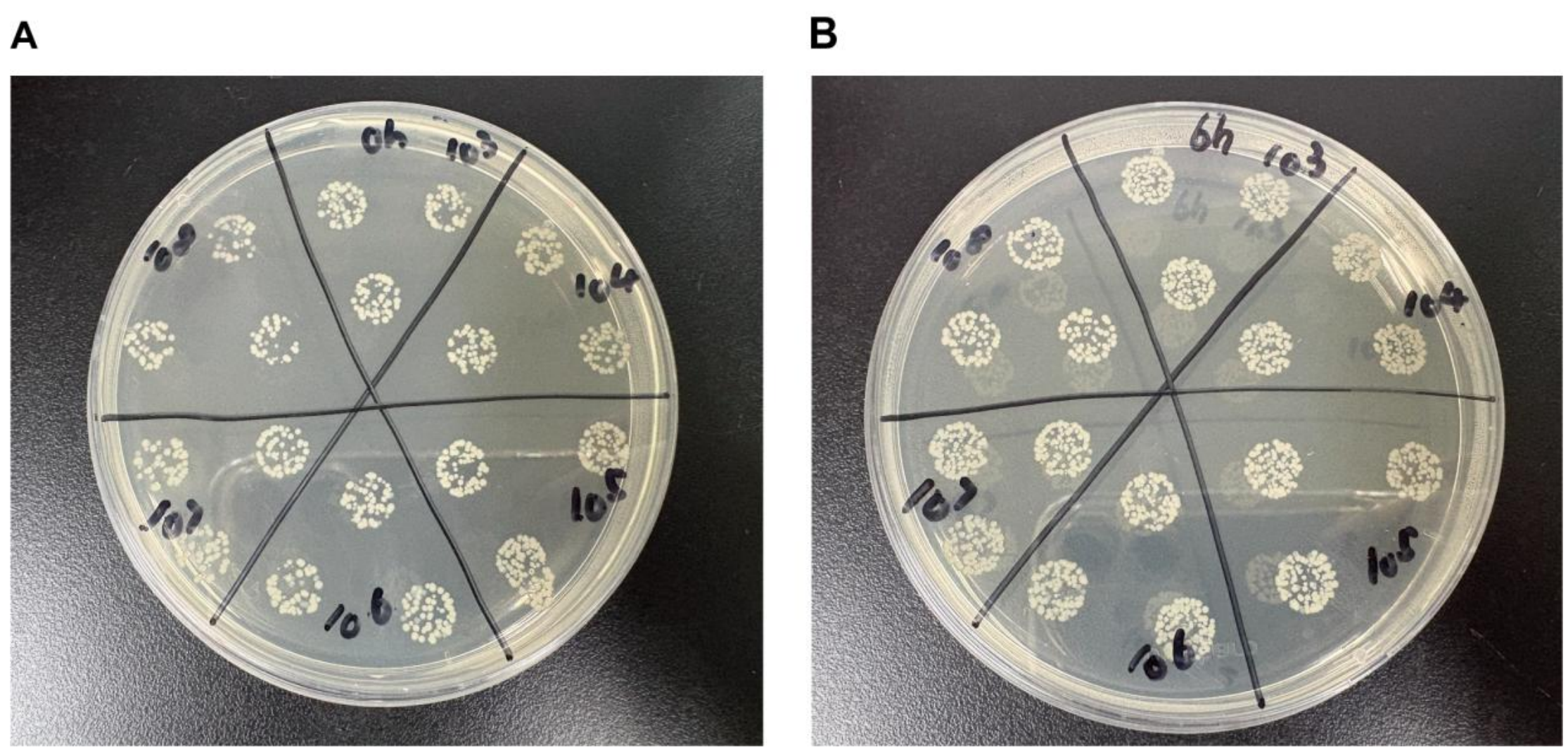

Supplementary Figure S7. Representative LB agar plates used for CFU enumeration under nutrient-limited conditions.

(A) Representative serial-dilution spot plates from *A. baumannii* cultured in 0.1% LB without antibiotics after 2 h of nutrient adaptation. **(B)** Representative serial-dilution spot plates from the corresponding 0.1% LB culture after an additional 6 h of antibiotic-free incubation. Serial dilutions are indicated on the plates. These plates were used for manual colony counting and representative CFU/mL calculations shown in Supplementary Table S8. Each labeled sector represents a separate dilution of the corresponding sample.

2 Supplementary Tables

Supplementary Table S1. MIC values of *A. baumannii* ATCC 19606^T for the antibiotics used in the main ATP–survival and MDI analyses.

Strain	Meropenem (µg/mL)	Ceftazidime (µg/mL)	Ciprofloxacin (µg/mL)	Tigecycline (µg/mL)	Amikacin (µg/mL)	Polymyxin B (µg/mL)
A. baumannii ATCC 19606^T	1	1	0.25	2	4	2

Supplementary Table S2. Maximum specific growth rates of *A. baumannii* ATCC 19606^T under different nutrient conditions.

LB concentration	µmax, Bio 1 (h^{-1})	µmax, Bio 2 (h^{-1})	µmax, Bio 3 (h^{-1})	Mean ± SEM (h^{-1})
100%	1.381	1.355	1.339	1.358 ± 0.012
10%	0.834	0.854	0.805	0.831 ± 0.014
1%	ND	ND	ND	ND
0.1%	ND	ND	ND	ND
0%	ND	ND	ND	ND

Note: ND, not reliably determinable because no qualifying exponential-growth interval meeting the predefined criteria was identified.

Supplementary Table S3. Primer sequence information.

Primer	Sequence (5′–3′)
Ab-spoT-F	TCGAAAAAGTGCTTGCTGCC
Ab-spoT-R	AGCTAACCGCGATAGGATGC
Ab-relA-F	CAACCAGTCACGGTTAGGCT
Ab-relA-R	GCGTGTTAACCAGCGCTATG
Ab-relE-F	GAGTGATGAAGAGTATCG
Ab-relE-R	CACCACCATTCTTGATTA
Ab-relB-F	CAGGTTCGACTTGACTCCCA
Ab-relB-R	CTGCTTGGGCAGGGGTAATC
Ab-hth-F	ATCACGGCGGTTTTACAGGT
Ab-hth-R	CACGTTCTGCATCGTAAGCG

Primer	Sequence (5′–3′)
Ab-gnat-F	GGAGGTTGTGAGTGATCT
Ab-gnat-R	ATGATGCTGAGGATGATGA
Ab-recA-F	ACCAATCCCCAATGCGATGT
Ab-recA-R	GCCGCTTTGAGCCAAATTGA
Ab-16S-F	TCTACTAGCCGTTGGAGCCT
Ab-16S-R	CCAGGTAAGGTTCTTCGCGT

Supplementary Table S4. Clinical isolate information and antimicrobial susceptibility testing results.

Sample ID	Sample Source	Identification	Meropenem AST	Amikacin AST
A. baumannii ATCC 19606^T	ATCC	/	+	+
A. baumannii 5993	Sputum	VITEK® 2 Compact	+	+
A. baumannii 5938	Venous Catheter Tip	VITEK® 2 Compact	+	+
A. baumannii 6000	Sputum	VITEK® 2 Compact	+	+
A. baumannii 8508	Sputum	VITEK® 2 Compact	+	+
A. baumannii 8922	Sputum	VITEK® 2 Compact	+	+

Note: "+" indicates susceptibility to the corresponding antibiotic based on antimicrobial susceptibility testing (AST). "/" indicates not applicable.

Supplementary Table S5. Dose-specific ATP–survival linear regression statistics and Metabolic Dependency Index (MDI) values.

Supplementary Table S5a. Linear regression statistics for meropenem (related to Figure 2A and 2H).

MEM Conc. (× MIC)	Slope (*k*)	95% CI	R²	Pearson *r*	*p* value
64× MIC	-245779	[-735968, 244410]	0.4591	-0.6776	0.2088
32× MIC	-209749	[-481816, 62318]	0.6674	-0.8169	0.0914
16× MIC	-201167	[-695252, 292919]	0.3588	-0.5990	0.2858
8× MIC	-180544	[-640749, 279662]	0.3419	-0.5847	0.3004
4× MIC	-193457	[-683601, 296688]	0.3447	-0.5871	0.2980
2× MIC	-180618	[-670821, 309584]	0.3143	-0.5606	0.3256
1× MIC	-135184	[-514999, 244631]	0.2996	-0.5473	0.3397
0.5× MIC	2459	[-474418, 479335]	0.0001	0.0095	0.9879
MDI (mean |*k*|)			168619		
Bootstrap 95% CI			[147546, 233242]		

Supplementary Table S5b. Linear regression statistics for tigecycline (related to Figure 2B and 2H).

TGC Conc. (× MIC)	Slope (*k*)	95% CI	R²	Pearson *r*	*p* value
64× MIC	-245779	[-735968, 244410]	0.4591	-0.6776	0.2088
32× MIC	-270748	[-470038, -71458]	0.8617	-0.9283	0.0228
16× MIC	-176855	[-232229, -121481]	0.9718	-0.9858	0.0020
8× MIC	-157907	[-369157, 53342]	0.6535	-0.8084	0.0977
4× MIC	-125337	[-356579, 105904]	0.4979	-0.7057	0.1830
2× MIC	-112198	[-354837, 130440]	0.4192	-0.6475	0.2375
1× MIC	-106416	[-376620, 163789]	0.3437	-0.5862	0.2989
0.5× MIC	-6835	[-240461, 226791]	0.0029	-0.0537	0.9317
MDI (mean |*k*|)			150260		
Bootstrap 95% CI			[121869, 166651]		

Supplementary Table S5c. Linear regression statistics for ceftazidime (related to Figure 2C and 2H).

CAZ Conc. (× MIC)	Slope (*k*)	95% CI	R²	Pearson *r*	*p* value
64× MIC	-64125	[-258366, 130116]	0.2690	-0.5186	0.3706
32× MIC	-67306	[-331906, 197294]	0.1793	-0.4234	0.4775
16× MIC	-53061	[-250620, 144498]	0.1958	-0.4425	0.4555
8× MIC	-51510	[-378432, 275412]	0.0773	-0.2781	0.6506
4× MIC	137004	[-80600, 354607]	0.5723	0.7565	0.1388
2× MIC	133656	[-136452, 403765]	0.4525	0.6727	0.2134
1× MIC	156856	[-177197, 490909]	0.4267	0.6532	0.2319
0.5× MIC	189828	[-6034, 385690]	0.7603	0.8719	0.0539
MDI (mean |*k*|)			106668		
Bootstrap 95% CI			[93948, 122726]		

Supplementary Table S5d. Linear regression statistics for amikacin (related to Figure 2D and 2H).

AMK Conc. (× MIC)	Slope (*k*)	95% CI	R²	Pearson *r*	*p* value
64× MIC	-11023	[-81458, 59413]	0.0764	-0.2763	0.6527
32× MIC	-11023	[-81458, 59413]	0.0764	-0.2763	0.6527
16× MIC	-11023	[-81458, 59413]	0.0764	-0.2763	0.6527
8× MIC	-11023	[-81458, 59413]	0.0764	-0.2763	0.6527
4× MIC	-11023	[-81458, 59413]	0.0764	-0.2763	0.6527
2× MIC	-37401	[-187386, 112585]	0.1735	-0.4165	0.4854
1× MIC	99842	[-112246, 311929]	0.4280	0.6542	0.2310
0.5× MIC	245097	[-41884, 532078]	0.7112	0.8433	0.0727
MDI (mean |*k*|)			54682		
Bootstrap 95% CI			[51576, 61452]		

Supplementary Table S5e. Linear regression statistics for polymyxin B (related to Figure 2E and 2H).

PMB Conc. (× MIC)	Slope (*k*)	95% CI	R^2	Pearson *r*	*p* value
64× MIC	-11023	[-81458, 59413]	0.0764	-0.2763	0.6527
32× MIC	-11023	[-81458, 59413]	0.0764	-0.2763	0.6527
16× MIC	-11023	[-81458, 59413]	0.0764	-0.2763	0.6527
8× MIC	-53953	[-657609, 549702]	0.0263	-0.1621	0.7946
4× MIC	-235936	[-883305, 411432]	0.3096	-0.5564	0.3300
2× MIC	139789	[-264446, 544023]	0.2876	0.5363	0.3515
1× MIC	138227	[-203941, 480396]	0.3552	0.5960	0.2888
0.5× MIC	120165	[-205711, 446041]	0.3146	0.5609	0.3253
MDI (mean |*k*|)			90142		
Bootstrap 95% CI			[72833, 106633]		

Supplementary Table S5f. Linear regression statistics for ciprofloxacin (related to Figure 2F and 2H).

CIP Conc. (× MIC)	Slope (*k*)	95% CI	R^2	Pearson *r*	*p* value
64× MIC	20946	[-364622, 406514]	0.0099	0.0993	0.8737
32× MIC	-10400	[-381027, 360227]	0.0027	0.0515	0.9345
16× MIC	-90453	[-488433, 307527]	0.1485	0.3854	0.5218
8× MIC	-121673	[-539693, 296347]	0.2224	0.4716	0.4226
4× MIC	-201021	[-708907, 306866]	0.3459	0.5882	0.2969
2× MIC	-140934	[-668087, 386218]	0.1944	0.4409	0.4574
1× MIC	81513	[-51473, 214499]	0.5592	0.7478	0.1462
0.5× MIC	106107	[-175603, 387816]	0.3238	0.5691	0.3167
MDI (mean |*k*|)			96631		
Bootstrap 95% CI			[74463, 103009]		

Note: Slopes, Pearson correlation coefficients, and *p* values were calculated using $\log_{10}$ survival. Therefore, the signs of the slopes and Pearson *r* values are opposite to those displayed in Figure 2A–F, in which $-\log_{10}$ survival was used for graphical presentation. MDI was calculated as the arithmetic mean of the eight absolute dose-specific slopes.

Supplementary Table S6. Replicate-level CFU counts underlying the antibiotic combination and staggered-treatment analyses.

Supplementary Table S6a. Replicate-level CFU counts following MEM-first staggered MEM–AMK treatment under nutrient-limited conditions (related to Figure 3A).

**AMK ↓ MEM → (µg/mL)**	**128**	**64**	**32**	**16**	**8**	**4**	**2**	**1**	**0**
	NCD	NCD	NCD	NCD	NCD	NCD	NCD	NCD	NCD
**32**	NCD	NCD	NCD	NCD	NCD	NCD	NCD	NCD	NCD
	NCD	NCD	NCD	NCD	NCD	NCD	NCD	NCD	NCD
	NCD	NCD	NCD	NCD	NCD	NCD	NCD	NCD	NCD
**16**	NCD	NCD	NCD	NCD	NCD	NCD	NCD	NCD	NCD
	NCD	NCD	NCD	NCD	NCD	NCD	NCD	NCD	NCD
	NCD	NCD	NCD	NCD	NCD	NCD	NCD	NCD	5.00E+02
**8**	NCD	NCD	NCD	NCD	NCD	NCD	NCD	NCD	5.00E+02
	NCD	NCD	NCD	NCD	NCD	NCD	NCD	NCD	5.00E+02
	4.00E+03	1.00E+03	3.00E+03	2.00E+03	2.00E+03	1.00E+03	5.00E+02	2.00E+03	1.00E+04
**4**	4.50E+03	1.50E+03	1.00E+03	2.00E+03	2.00E+03	1.50E+03	5.00E+02	1.50E+03	1.50E+04
	5.00E+03	2.00E+03	4.50E+03	2.50E+03	2.00E+03	2.00E+03	5.00E+02	5.00E+02	2.00E+04
	1.00E+04	1.10E+04	1.00E+04	1.00E+03	2.50E+06	4.50E+04	7.00E+02	1.00E+05	1.00E+06
**2**	5.00E+03	8.50E+03	2.50E+04	2.50E+03	1.00E+06	4.50E+04	5.50E+02	2.00E+05	1.00E+06
	1.50E+04	5.00E+03	3.00E+04	2.00E+03	1.00E+06	4.00E+04	6.50E+02	1.00E+05	1.50E+06
	1.50E+06	2.00E+06	1.40E+06	1.20E+06	3.00E+06	8.50E+05	3.50E+05	5.50E+05	2.50E+05
**1**	3.00E+06	4.50E+06	1.50E+06	1.20E+06	2.50E+06	7.50E+05	3.00E+05	3.00E+05	5.00E+04
	4.50E+06	4.50E+06	2.15E+06	1.05E+06	4.50E+06	9.50E+05	6.00E+05	4.50E+05	3.00E+05
	8.50E+10	9.00E+06	5.50E+07	8.50E+08	2.50E+06	9.50E+06	1.50E+06	9.50E+05	5.00E+06
**0.5**	6.50E+10	5.00E+06	4.50E+07	1.35E+09	3.50E+06	1.40E+07	3.00E+06	1.35E+06	9.00E+06
	6.50E+10	8.50E+06	4.00E+07	1.30E+09	4.50E+06	1.45E+07	4.00E+06	1.10E+06	1.05E+07
	9.50E+08	1.50E+07	1.00E+06	1.05E+06	2.00E+06	1.00E+07	4.50E+08	5.00E+07	1.00E+09
**0.25**	1.05E+09	5.50E+07	1.50E+06	9.00E+05	6.50E+06	1.35E+07	6.50E+09	4.50E+07	5.00E+08
	1.05E+09	3.50E+07	1.00E+06	1.35E+06	5.00E+06	1.35E+07	6.50E+08	5.00E+07	5.00E+08
	4.50E+10	9.50E+09	7.00E+08	4.00E+10	7.50E+10	8.50E+10	1.00E+10	7.00E+10	1.00E+10
**0**	7.00E+10	7.50E+09	6.00E+08	8.50E+10	7.00E+10	1.25E+10	7.50E+09	5.50E+10	1.50E+10
	5.00E+10	9.50E+09	3.50E+08	6.50E+10	7.50E+10	1.30E+10	9.50E+09	6.00E+10	5.00E+10

Supplementary Table S6b. Replicate-level CFU counts following simultaneous MEM–AMK treatment under nutrient-limited conditions (related to Figure 3B).

**AMK ↓ MEM → (µg/mL)**	**128**	**64**	**32**	**16**	**8**	**4**	**2**	**1**	**0**
	NCD	NCD	NCD	NCD	NCD	NCD	NCD	NCD	NCD
**32**	NCD	NCD	NCD	NCD	NCD	NCD	NCD	NCD	NCD
	NCD	NCD	NCD	NCD	NCD	NCD	NCD	NCD	NCD
	NCD	NCD	NCD	NCD	NCD	NCD	NCD	NCD	NCD
**16**	NCD	NCD	NCD	NCD	NCD	NCD	NCD	NCD	NCD
	NCD	NCD	NCD	NCD	NCD	NCD	NCD	NCD	NCD
	NCD	NCD	NCD	NCD	NCD	NCD	NCD	NCD	1.00E+03
8	NCD	NCD	NCD	NCD	NCD	NCD	NCD	NCD	5.00E+02
	NCD	NCD	NCD	NCD	NCD	NCD	NCD	NCD	1.00E+03
	NCD	1.00E+03	NCD	1.50E+03	1.00E+03	NCD	1.00E+03	5.00E+02	1.00E+04
4	NCD	5.00E+02	NCD	1.00E+03	1.00E+03	NCD	1.00E+03	5.00E+02	1.50E+04
	NCD	5.00E+02	NCD	1.50E+03	5.00E+02	NCD	1.00E+03	1.00E+03	2.00E+04
	4.00E+03	4.50E+04	7.50E+04	3.50E+04	4.00E+04	1.50E+04	1.50E+04	1.50E+04	5.00E+05
2	8.00E+03	3.50E+04	5.50E+04	3.00E+04	5.50E+04	2.50E+04	4.00E+04	3.00E+04	5.00E+05
	5.00E+03	3.50E+04	5.00E+04	2.00E+04	3.50E+04	3.00E+04	2.50E+04	3.00E+04	5.00E+05
	1.00E+07	6.00E+06	7.00E+06	6.00E+06	1.10E+07	1.15E+07	1.50E+07	7.50E+06	5.00E+06
1	4.50E+06	8.50E+06	6.00E+06	9.00E+06	1.40E+07	2.35E+07	6.50E+06	4.00E+06	1.00E+07
	3.50E+06	8.00E+06	9.50E+06	1.10E+07	1.05E+07	1.50E+07	1.40E+07	3.50E+06	5.00E+06
	1.25E+07	1.65E+07	2.00E+08	6.00E+08	2.50E+08	2.00E+08	5.00E+07	5.00E+08	5.00E+06
0.5	1.50E+07	1.25E+07	1.50E+08	3.00E+08	2.00E+08	1.00E+08	6.00E+07	1.50E+09	5.00E+06
	1.25E+07	1.60E+07	5.00E+07	5.00E+08	5.00E+07	1.00E+08	8.50E+07	5.00E+08	5.00E+06
	1.40E+09	9.50E+08	9.50E+08	4.00E+09	7.00E+08	6.50E+08	6.00E+09	6.00E+10	5.00E+08
0.25	1.60E+09	1.15E+09	1.25E+09	7.00E+09	6.50E+08	7.00E+08	9.00E+09	5.00E+10	5.00E+08
	1.25E+09	1.50E+09	1.30E+09	9.50E+09	6.50E+08	7.00E+08	7.00E+09	7.50E+10	5.00E+08
	2.25E+10	2.95E+10	6.00E+10	1.90E+10	7.50E+10	2.40E+10	1.60E+10	1.50E+10	7.00E+10
0	2.40E+10	2.25E+10	9.00E+10	2.05E+10	7.00E+10	1.25E+10	1.90E+10	1.40E+10	4.50E+10
	2.30E+10	2.10E+10	8.00E+10	2.30E+10	7.50E+10	1.30E+10	1.75E+10	1.30E+10	7.00E+10

Supplementary Table S6c. Replicate-level CFU counts from the MEM–PMB checkerboard killing assay under nutrient-limited conditions (related to Figure 3C).

**PMB ↓ MEM → (µg/mL)**	**128**	**64**	**32**	**16**	**8**	**4**	**0**
	8.00E+03	7.00E+03	1.00E+04	1.00E+04	1.45E+04	5.00E+03	1.70E+08
**32**	9.50E+03	6.00E+03	1.00E+04	1.50E+04	1.45E+04	1.00E+04	1.60E+08
	7.00E+03	9.50E+03	1.50E+04	1.00E+04	1.45E+04	1.00E+04	1.55E+08
	5.50E+05	6.00E+06	1.10E+06	7.00E+05	1.50E+06	4.50E+06	5.00E+10
**16**	4.00E+05	2.50E+06	3.50E+05	2.50E+05	2.50E+06	5.50E+06	5.00E+10
	3.00E+05	2.50E+06	3.00E+05	4.00E+05	4.00E+06	3.50E+06	5.00E+10
	2.00E+06	1.50E+07	1.50E+07	1.50E+08	5.00E+07	5.00E+07	1.40E+10
**8**	1.50E+06	2.00E+07	1.50E+07	2.00E+08	5.00E+07	5.00E+07	1.25E+10
	5.00E+06	2.00E+07	1.50E+07	4.00E+08	1.00E+08	5.00E+07	1.15E+10
	1.40E+09	2.00E+10	1.10E+09	8.00E+09	1.95E+10	2.30E+10	8.50E+09
**4**	9.50E+08	2.15E+10	1.10E+09	8.00E+09	1.40E+10	1.55E+10	1.05E+10
	1.50E+09	1.70E+10	9.50E+08	7.50E+09	1.55E+10	1.40E+10	9.00E+09
	1.00E+10	5.50E+10	5.00E+09	1.00E+10	7.00E+08	5.50E+10	2.50E+10
**0**	3.00E+10	6.50E+10	5.00E+09	1.50E+10	9.00E+08	6.50E+10	1.50E+10
	2.50E+10	7.00E+10	5.00E+09	1.50E+10	6.50E+08	6.00E+10	1.50E+10

Supplementary Table S6d. Replicate-level CFU counts from the AMK–PMB checkerboard killing assay under nutrient-limited conditions (related to Figure 3D).

**AMK ↓ PMB → (μg/mL)**	**8**	**4**	**2**	**1**	**0**
	1.50E+04	2.00E+04	4.00E+04	1.00E+09	5.00E+10
**8**	2.00E+04	3.50E+04	4.50E+04	5.00E+08	5.00E+10
	2.50E+04	2.50E+04	8.50E+04	5.00E+08	2.00E+10
	7.50E+04	8.50E+04	6.50E+04	3.50E+09	1.05E+10
**4**	1.00E+05	7.50E+04	6.50E+04	4.00E+09	1.65E+10
	1.00E+05	7.00E+04	5.50E+04	3.50E+09	1.20E+10
	4.00E+04	1.30E+06	1.10E+09	1.45E+11	7.50E+10
**2**	6.00E+04	1.35E+06	1.55E+09	1.85E+11	6.50E+10
	7.00E+04	1.05E+06	9.50E+08	1.30E+11	7.00E+10
	NCD	6.00E+04	1.00E+07	1.95E+10	5.00E+10
**1**	NCD	6.50E+04	1.00E+07	1.20E+10	5.00E+10
	NCD	6.50E+04	1.00E+07	1.05E+10	5.00E+10
	NCD	5.50E+05	9.00E+06	7.50E+10	3.00E+10
**0.5**	NCD	6.50E+05	8.50E+06	7.50E+10	4.00E+10
	NCD	6.50E+05	1.35E+07	7.50E+10	4.00E+10
	NCD	1.15E+04	9.50E+06	2.50E+10	4.50E+10
**0.25**	NCD	1.15E+04	1.25E+07	2.50E+10	5.50E+10
	NCD	9.00E+03	7.50E+06	2.00E+10	4.00E+10
	5.00E+06	5.00E+07	1.60E+08	1.20E+10	1.50E+10
**0**	5.00E+06	5.00E+07	1.90E+08	1.20E+10	5.00E+10
	5.00E+06	5.00E+07	1.95E+08	1.35E+10	1.50E+10

Supplementary Table S6e. Replicate-level CFU counts from the MEM–TGC checkerboard killing assay under nutrient-limited conditions (related to Figure 3E).

**MEM ↓ TGC → (μg/mL)**	**128**	**64**	**32**	**16**	**8**	**4**	**0**
	3.00E+07	2.50E+08	7.50E+08	5.00E+08	2.50E+07	1.30E+08	5.00E+09
**32**	5.00E+07	2.50E+08	5.50E+08	3.00E+08	2.50E+07	9.50E+07	5.00E+09
	6.00E+07	2.50E+08	9.50E+08	2.00E+08	6.00E+07	9.00E+07	5.00E+09
	7.50E+08	1.25E+10	5.00E+10	8.00E+10	5.00E+10	1.00E+10	1.35E+10
**16**	7.00E+08	1.10E+10	5.00E+10	8.00E+10	8.00E+10	5.00E+09	1.10E+10
	8.50E+08	1.15E+10	5.00E+10	6.00E+10	1.25E+10	5.00E+09	1.15E+10
	6.00E+07	2.50E+07	4.50E+07	1.00E+07	1.50E+07	1.00E+07	2.50E+10
**8**	6.00E+07	3.00E+07	3.00E+07	2.50E+07	1.50E+07	1.00E+07	1.50E+10
	4.00E+07	2.50E+07	3.00E+07	4.00E+07	3.00E+07	1.00E+07	3.00E+10
	1.50E+08	9.50E+07	7.00E+08	3.50E+08	2.00E+07	2.50E+09	3.50E+10
**4**	5.00E+07	9.50E+07	3.00E+08	2.50E+08	2.50E+07	3.50E+09	2.50E+10
	1.00E+08	7.00E+07	5.00E+08	3.00E+08	3.00E+07	7.50E+09	3.00E+10
	2.50E+10	1.55E+10	6.00E+09	8.00E+10	6.50E+08	3.00E+09	3.00E+10
**2**	5.00E+09	1.50E+10	6.00E+09	7.00E+10	3.00E+08	3.00E+09	5.00E+10
	1.50E+10	1.65E+10	5.50E+09	3.00E+10	5.00E+08	2.50E+09	7.00E+10
	1.50E+10	1.15E+09	1.80E+09	1.50E+09	1.70E+09	7.00E+08	4.00E+10
**1**	1.00E+10	1.25E+09	1.05E+09	1.50E+09	1.40E+09	6.50E+08	3.50E+10
	1.00E+10	1.20E+09	1.35E+09	1.85E+09	1.45E+09	7.00E+08	6.00E+10
	1.50E+07	4.50E+07	1.50E+07	4.50E+07	1.00E+07	5.50E+07	2.50E+10
**0**	2.00E+07	5.00E+07	1.50E+07	2.50E+07	4.00E+07	3.50E+07	3.50E+10
	2.50E+07	2.50E+07	3.00E+07	3.50E+07	4.00E+07	4.00E+07	6.00E+10

Supplementary Table S6f. Replicate-level CFU counts from the MEM–TGC checkerboard killing assay under nutrient-limited conditions (related to Figure 3F).

**MEM ↓ TGC → (µg/mL)**	**32**	**8**	**2**	**0.5**	**0**
	1.50E+07	2.00E+12	2.00E+10	5.50E+07	1.50E+07
**128**	1.50E+07	5.00E+12	2.00E+10	1.00E+08	2.00E+07
	2.00E+07	8.00E+12	3.50E+10	8.00E+07	2.50E+07
	5.00E+07	6.50E+07	6.50E+12	3.00E+09	1.50E+07
**32**	5.00E+07	5.00E+07	6.50E+12	3.50E+09	1.50E+07
	1.00E+08	6.00E+07	1.05E+12	3.00E+09	3.00E+07
	5.00E+08	6.00E+08	5.00E+08	3.50E+09	5.00E+07
**8**	5.00E+08	3.50E+09	2.00E+09	5.00E+09	5.00E+07
	5.00E+08	4.50E+07	2.00E+09	7.00E+09	5.00E+07
	1.50E+09	5.00E+09	5.00E+10	7.00E+07	1.50E+08
**4**	3.00E+09	1.00E+10	5.00E+10	5.50E+07	1.00E+08
	3.00E+09	5.00E+09	1.50E+11	1.15E+08	1.50E+08
	2.00E+10	1.35E+10	3.00E+10	1.45E+10	2.50E+10
**0**	4.50E+10	1.10E+10	5.00E+10	1.50E+10	3.50E+10
	5.50E+10	1.15E+10	7.00E+10	1.80E+10	6.00E+10

Supplementary Table S6g. Replicate-level CFU counts following reverse-order AMK-first staggered MEM–AMK treatment under nutrient-limited conditions (related to Supplementary Figure S5).

**AMK ↓ MEM → (µg/mL)**	**128**	**64**	**32**	**16**	**8**	**4**	**2**	**1**	**0**
	NCD	NCD	NCD	NCD	NCD	NCD	NCD	NCD	NCD
**32**	NCD	NCD	NCD	NCD	NCD	NCD	NCD	NCD	NCD
	NCD	NCD	NCD	NCD	NCD	NCD	NCD	NCD	NCD
	NCD	NCD	NCD	NCD	NCD	NCD	NCD	NCD	NCD
**16**	NCD	NCD	NCD	NCD	NCD	NCD	NCD	NCD	NCD
	NCD	NCD	NCD	NCD	NCD	NCD	NCD	NCD	NCD
	NCD	NCD	NCD	NCD	NCD	NCD	NCD	NCD	NCD
**8**	NCD	NCD	NCD	NCD	NCD	NCD	NCD	NCD	NCD
	NCD	NCD	NCD	NCD	NCD	NCD	NCD	NCD	NCD
	NCD	NCD	NCD	NCD	NCD	NCD	NCD	5.00E+03	1.10E+07
**4**	NCD	NCD	NCD	NCD	NCD	NCD	NCD	5.00E+03	9.50E+06
	NCD	NCD	NCD	NCD	NCD	NCD	NCD	5.00E+03	9.00E+06
	1.50E+04	1.00E+05	1.50E+06	1.00E+06	1.00E+06	3.00E+07	1.50E+07	2.50E+07	2.50E+08
**2**	2.50E+04	5.00E+04	1.00E+06	1.50E+06	1.50E+06	3.50E+07	1.00E+07	2.50E+07	1.00E+08
	2.50E+04	5.00E+04	2.00E+06	1.50E+06	1.50E+06	3.50E+07	1.50E+07	5.00E+06	1.50E+08
	5.00E+06	1.50E+07	2.00E+07	1.50E+07	1.50E+08	2.50E+08	1.50E+08	1.00E+08	2.50E+09
**1**	5.00E+06	2.00E+07	1.50E+07	1.00E+07	2.50E+08	3.50E+07	1.50E+08	5.00E+07	5.00E+08
	1.50E+07	5.00E+07	3.50E+07	5.00E+06	5.00E+07	3.50E+07	1.00E+08	5.00E+07	5.00E+08
	1.00E+06	1.50E+06	1.50E+07	1.50E+07	5.00E+06	1.50E+07	1.00E+09	1.00E+08	5.00E+07
**0.5**	1.00E+06	1.50E+06	2.50E+07	5.00E+06	5.00E+06	1.50E+07	5.00E+08	5.00E+07	5.00E+07
	1.00E+06	5.00E+05	2.50E+07	5.00E+06	3.00E+07	1.50E+07	1.00E+09	5.00E+07	1.00E+08
	5.00E+04	1.00E+07	1.50E+09	1.50E+09	3.00E+09	1.00E+09	3.50E+09	1.00E+10	2.50E+09
**0.25**	2.50E+05	1.00E+07	2.50E+09	2.50E+09	2.50E+09	1.50E+09	4.50E+09	5.00E+09	5.00E+08
	2.50E+05	1.50E+07	5.00E+08	1.50E+09	2.50E+09	2.50E+09	4.50E+09	5.00E+09	5.00E+08
	1.50E+09	1.50E+10	2.50E+10	1.00E+10	2.00E+10	1.00E+10	1.50E+10	1.50E+10	1.40E+11
**0**	2.50E+09	2.00E+10	2.00E+10	1.00E+10	2.50E+10	1.00E+10	1.00E+10	5.00E+10	1.35E+11
	5.00E+08	1.00E+10	1.50E+10	1.00E+10	5.00E+10	1.50E+10	5.00E+10	5.00E+10	1.45E+11

Note: Each set of three CFU/mL values represents three independent biological replicates. NCD, no colonies detected under the plating conditions used. NCD values were not assigned a numerical CFU value and do not represent 0 CFU/mL.

Supplementary Table S7. Replicate-level CFU counts following staggered and simultaneous MEM–AMK treatment of clinical *A. baumannii* isolates.

Supplementary Table S7a. MEM-first staggered MEM–AMK treatment.

***A. baumannii* 5993**			***A. baumannii* 5938**			***A. baumannii* 6000**			***A. baumannii* 8508**			***A. baumannii* 8922**		
**MEM (µg/mL)**	**AMK (µg/mL)**	**CFU/mL**	**MEM (µg/mL)**	**AMK (µg/mL)**	**CFU/mL**	**MEM (µg/mL)**	**AMK (µg/mL)**	**CFU/mL**	**MEM (µg/mL)**	**AMK (µg/mL)**	**CFU/mL**	**MEM (µg/mL)**	**AMK (µg/mL)**	**CFU/mL**
		5.00E+10			5.00E+08			3.00E+10			1.00E+10			5.00E+09
–	–	5.00E+10	–	–	5.00E+08	–	–	2.00E+10	–	–	3.00E+10	–	–	3.00E+10
		5.00E+10			5.00E+08			5.00E+09			1.00E+10			5.00E+09
		1.00E+09			1.00E+08			3.50E+09			1.30E+10			1.30E+10
128	–	2.50E+09	128	–	1.00E+08	128	–	4.50E+09	128	–	1.30E+10	128	–	1.70E+10
		2.50E+09			1.00E+08			4.00E+09			1.65E+10			1.30E+10
		2.50E+10			5.00E+09			2.50E+09			8.50E+10			4.50E+11
2	–	2.50E+10	2	–	5.00E+09	2	–	1.00E+09	2	–	6.00E+10	2	–	5.00E+11
		2.50E+10			5.00E+09			1.50E+09			7.50E+10			4.50E+11
		NCD			NCD			NCD			NCD			NCD
–	32	NCD	–	32	NCD	–	32	NCD	–	32	NCD	–	32	NCD
		NCD			NCD			NCD			NCD			NCD
		NCD			NCD			NCD			NCD			NCD
–	16	NCD	–	16	NCD	–	16	NCD	–	16	NCD	–	16	NCD
		NCD			NCD			NCD			NCD			NCD
		NCD			NCD			NCD			NCD			NCD
–	8	NCD	–	8	NCD	–	8	NCD	–	8	NCD	–	8	NCD
		NCD			NCD			NCD			NCD			NCD
		NCD			5.00E+02			NCD			5.00E+06			1.00E+06
–	4	NCD	–	4	NCD	–	4	NCD	–	4	5.00E+06	–	4	1.00E+06
		NCD			5.00E+02			NCD			5.00E+06			1.00E+06
		5.00E+06			5.00E+04			1.00E+06			5.00E+08			3.50E+10
–	1	5.00E+06	–	1	5.00E+04	–	1	7.50E+05	–	1	5.00E+08	–	1	2.00E+10
		5.00E+06			5.00E+04			7.50E+05			5.00E+08			1.00E+10
		NCD			NCD			NCD			NCD			NCD
128	32	NCD	128	32	NCD	128	32	NCD	128	32	NCD	128	32	NCD
		NCD			NCD			NCD			NCD			NCD
		NCD			NCD			NCD			NCD			NCD
128	16	NCD	128	16	NCD	128	16	NCD	128	16	NCD	128	16	NCD
		NCD			NCD			NCD			NCD			NCD
		NCD			NCD			NCD			NCD			NCD
128	8	NCD	128	8	NCD	128	8	NCD	128	8	NCD	128	8	NCD
		NCD			NCD			NCD			NCD			NCD
		NCD			NCD			NCD			1.00E+07			5.00E+01
128	4	NCD	128	4	NCD	128	4	NCD	128	4	1.00E+07	128	4	1.50E+02
		NCD			NCD			NCD			1.00E+07			1.50E+02
		1.00E+06			5.00E+05			1.50E+05			5.00E+06			1.00E+08
128	1	5.00E+05	128	1	5.00E+05	128	1	1.50E+05	128	1	5.00E+06	128	1	1.00E+08
		5.00E+05			5.00E+05			1.50E+05			5.00E+06			2.00E+08
2	32	NCD	2	32	NCD	2	32	NCD	2	32	NCD	2	32	NCD
		NCD			NCD			NCD			NCD			NCD

A. baumannii 5993			*A. baumannii* 5938			*A. baumannii* 6000			*A. baumannii* 8508			*A. baumannii* 8922		
**MEM (µg/mL)**	**AMK (µg/mL)**	**CFU/mL**	**MEM (µg/mL)**	**AMK (µg/mL)**	**CFU/mL**	**MEM (µg/mL)**	**AMK (µg/mL)**	**CFU/mL**	**MEM (µg/mL)**	**AMK (µg/mL)**	**CFU/mL**	**MEM (µg/mL)**	**AMK (µg/mL)**	**CFU/mL**
		NCD			NCD			NCD			NCD			NCD
		NCD			NCD			NCD			NCD			NCD
2	16	NCD	2	16	NCD	2	16	NCD	2	16	NCD	2	16	NCD
		NCD			NCD			NCD			NCD			NCD
		NCD			NCD			NCD			NCD			NCD
2	8	NCD	2	8	NCD	2	8	NCD	2	8	NCD	2	8	NCD
		NCD			NCD			NCD			NCD			NCD
		NCD			NCD			NCD			5.00E+06			1.50E+06
2	4	NCD	2	4	NCD	2	4	NCD	2	4	5.00E+06	2	4	1.50E+06
		NCD			NCD			NCD			5.00E+06			1.50E+06
		5.00E+06			5.00E+06			1.00E+05			5.00E+07			2.50E+09
2	1	5.00E+06	2	1	5.00E+06	2	1	1.50E+05	2	1	5.00E+07	2	1	2.50E+09
		5.00E+06			5.00E+06			5.00E+04			5.00E+07			3.00E+09

Supplementary Table S7b. Simultaneous MEM ‑ AMK treatment.

A. baumannii 5993			*A. baumannii* 5938			*A. baumannii* 6000			*A. baumannii* 8508			*A. baumannii* 8922		
**MEM (µg/mL)**	**AMK (µg/mL)**	**CFU/mL**	**MEM (µg/mL)**	**AMK (µg/mL)**	**CFU/mL**	**MEM (µg/mL)**	**AMK (µg/mL)**	**CFU/mL**	**MEM (µg/mL)**	**AMK (µg/mL)**	**CFU/mL**	**MEM (µg/mL)**	**AMK (µg/mL)**	**CFU/mL**
		1.50E+10			5.00E+10			1.00E+10			2.50E+11			1.00E+10
–	–	2.50E+10	–	–	5.00E+10	–	–	2.00E+10	–	–	2.00E+11	–	–	5.00E+09
		5.00E+9			5.00E+10			2.00E+10			3.50E+11			5.00E+09
		1.50E+10			1.50E+10			5.00E+09			1.45E+10			2.00E+10
128	–	1.50E+10	128	–	2.00E+10	128	–	1.00E+10	128	–	1.60E+10	128	–	3.00E+10
		5.00E+10			2.00E+10			1.50E+10			1.20E+10			4.00E+10
		7.00E+09			1.50E+09			8.00E+09			1.50E+10			3.50E+10
2	–	1.20E+10	2	–	5.00E+08	2	–	6.50E+09	2	–	1.00E+10	2	–	3.50E+10
		9.50E+09			3.50E+09			8.00E+09			2.00E+10			6.00E+10
		NCD			NCD			NCD			NCD			NCD
–	32	NCD	–	32	NCD	–	32	NCD	–	32	NCD	–	32	NCD
		NCD			NCD			NCD			NCD			NCD
		NCD			NCD			NCD			NCD			NCD
–	16	NCD	–	16	NCD	–	16	NCD	–	16	NCD	–	16	NCD
		NCD			NCD			NCD			NCD			NCD
		NCD			NCD			NCD			NCD			NCD
–	8	NCD	–	8	NCD	–	8	NCD	–	8	NCD	–	8	NCD
		NCD			NCD			NCD			NCD			NCD
		2.50E+03			2.00E+04			1.00E+05			5.00E+07			3.50E+05
–	4	1.50E+04	–	4	1.00E+04	–	4	1.00E+05	–	4	5.00E+07	–	4	4.00E+05
		1.50E+04			5.00E+04			1.00E+05			5.00E+07			3.50E+05
		1.00E+08			4.00E+07			1.50E+07			1.00E+10			5.00E+08
–	1	2.50E+08	–	1	3.50E+07	–	1	2.00E+07	–	1	1.00E+10	–	1	5.00E+08
		3.50E+08			5.00E+06			2.00E+07			2.00E+10			5.00E+08
		NCD			NCD			NCD			NCD			NCD
128	32	NCD	128	32	NCD	128	32	NCD	128	32	NCD	128	32	NCD
		NCD			NCD			NCD			NCD			NCD
		NCD			NCD			NCD			NCD			NCD
128	16	NCD	128	16	NCD	128	16	NCD	128	16	NCD	128	16	NCD
		NCD			NCD			NCD			NCD			NCD
		NCD			NCD			NCD			NCD			NCD
128	8	NCD	128	8	NCD	128	8	NCD	128	NCD	NCD	128	8	NCD
		NCD			NCD			NCD			NCD			NCD
		5.00E+01			2.00E+04			5.50E+05			1.00E+06			5.00E+06
128	4	5.00E+01	128	4	4.00E+04	128	4	5.50E+05	128	4	1.00E+06	128	4	7.00E+06
		5.00E+01			3.50E+04			5.50E+05			5.00E+05			5.00E+06
		1.50E+10			1.00E+09			5.00E+07			3.50E+10			4.50E+08
128	1	2.50E+10	128	1	5.00E+08	128	1	5.00E+07	128	1	2.00E+09	128	1	5.50E+08
		4.00E+10			1.00E+09			5.00E+07			4.50E+10			4.00E+08
		NCD			NCD			NCD			NCD			NCD
2	32	NCD	2	32	NCD	2	32	NCD	2	32	NCD	2	32	NCD
		NCD			NCD			NCD			NCD			NCD
		NCD			NCD			NCD			NCD			NCD
2	16	NCD	2	16	NCD	2	16	NCD	2	16	NCD	2	16	NCD
		NCD			NCD			NCD			NCD			NCD

A. baumannii 5993			*A. baumannii* 5938			*A. baumannii* 6000			*A. baumannii* 8508			*A. baumannii* 8922		
MEM (μg/mL)	AMK (μg/mL)	CFU/mL	MEM (μg/mL)	AMK (μg/mL)	CFU/mL	MEM (μg/mL)	AMK (μg/mL)	CFU/mL	MEM (μg/mL)	AMK (μg/mL)	CFU/mL	MEM (μg/mL)	AMK (μg/mL)	CFU/mL
		NCD			NCD			NCD			NCD			NCD
2	8	NCD	2	8	NCD	2	8	NCD	2	8	NCD	2	8	NCD
		NCD			NCD			NCD			NCD			NCD
		5.00E+06			5.00E+06			5.00E+02			2.50E+04			1.50E+04
2	4	4.00E+06	2	4	1.00E+05	2	4	1.50E+03	2	4	1.00E+04	2	4	1.50E+04
		3.50E+06			5.00E+04			5.00E+02			1.50E+04			1.35E+04
		5.50E+09			1.00E+05			1.00E+09			1.00E+10			5.50E+07
2	1	9.00E+09	2	1	5.00E+06	2	1	1.00E+09	2	1	5.00E+09	2	1	5.50E+07
		5.00E+09			1.00E+05			1.00E+09			1.00E+10			5.50E+07

Note: "–" indicates that the corresponding antibiotic was not added. Each set of three CFU/mL values represents three independent biological replicates. NCD, no colonies detected under the plating conditions used; NCD values were not assigned a numerical CFU value and do not represent 0 CFU/mL.

Supplementary Table S8. Verification of CFU/mL calculations under nutrient-limited conditions.

Condition	Adaptation time	OD_{600}	Original culture volume (μL)	Resuspension volume (μL)	Reciprocal dilution factor	Volume plated	Colony count	Calculation	Calculated CFU/mL
0.1% LB no antibiotic	2 h	0.1	200	100	10^8	10 μL	32	$32 \times 10^8 \times 50$	1.6×10^{11}
0.1% LB no antibiotic	2 h + 6 h	0.092	200	100	10^8	10 μL	52	$52 \times 10^8 \times 50$	2.6×10^{11}

Note: The entire 200-μL culture was collected by centrifugation and resuspended in 100 μL PBS. A 10-μL aliquot of the resuspended sample was serially diluted and spot-plated onto LB agar. For the representative plates shown, the reciprocal dilution factor was 10^8. Under these conditions, CFU/mL in the original culture was calculated as the manually counted colony number multiplied by the reciprocal dilution factor and a conversion factor of 50. Thus, the representative calculations were $32 \times 10^8 \times 50 = 1.6 \times 10^{11}$ CFU/mL and $52 \times 10^8 \times 50 = 2.6 \times 10^{11}$ CFU/mL. The values shown are representative examples based on manually counted colonies.

Supplementary Table S9. Sensitivity analysis of the Metabolic Dependency Index under alternative NCD pseudocounts.

Antibiotic	NCD handling	NCD pseudocount (CFU/mL)	MDI (mean |*k*|)	Rank
MEM	Primary analysis (NCD=1)	1	168619.46	1
TGC	Primary analysis (NCD=1)	1	150259.51	2
CAZ	Primary analysis (NCD=1)	1	106668.23	3
CIP	Primary analysis (NCD=1)	1	96630.83	4
PMB	Primary analysis (NCD=1)	1	90142.33	5
AMK	Primary analysis (NCD=1)	1	54681.57	6
MEM	LOD/2 (NCD=25)	25	168619.46	1
TGC	LOD/2 (NCD=25)	25	127986.18	2
CAZ	LOD/2 (NCD=25)	25	106668.22	3
CIP	LOD/2 (NCD=25)	25	95837.08	4
PMB	LOD/2 (NCD=25)	25	93075.98	5
AMK	LOD/2 (NCD=25)	25	50482.84	6
MEM	LOD (NCD=50)	50	168619.46	1
TGC	LOD (NCD=50)	50	125283.67	2
CAZ	LOD (NCD=50)	50	106668.22	3
CIP	LOD (NCD=50)	50	96251.87	4
PMB	LOD (NCD=50)	50	93075.98	5
AMK	LOD (NCD=50)	50	50288.71	6

Note: For the exploratory MDI sensitivity analyses, NCD observations were assigned pseudocounts of 1, 25, or 50 CFU/mL. For the present plating scheme, 50 CFU/mL was used as the assay detection-limit value; therefore, 25 CFU/mL represented LOD/2. These pseudocounts were used only for sensitivity analyses and do not represent measured CFU values.
